# Integrated single-cell TCR analysis across tissue identifies GZMK^+^CD8^+^ T cells promoting fibrosis via TGF-β signalling in IPF

**DOI:** 10.7150/ijbs.134974

**Published:** 2026-09-03

**Authors:** Bingqing Yue, Shuliang Jing, Qiaxuan Li, Guoyu Xie, Juan Chen, Tao Yan, Ganggui Zhu, Bin Huang, Jin Zhao, Xiucheng Yang, Jian Huang, Pan Yin, Qifeng Yao, Huijing Yang, Fei Gao, Jun Yang, Man Huang, Jingyu Chen

**Affiliations:** 1 Department of Lung Transplantation, Second Affiliated Hospital, Zhejiang University School of Medicine, 1511 Jianghong Road, Binjiang District, Hangzhou 310051, Zhejiang Province, China.; 2 Key Laboratory of Multiple Organ Failure (Zhejiang University), Ministry of Education, Hangzhou 310051, Zhejiang, China.; 3 Department of Physiology, Zhejiang University School of Medicine, State Key Laboratory of Transvascular Implantation Devices, 866 Yuhangtang Road, Hangzhou 310058, Zhejiang Province, China.; 4 Department of General Intensive Care Unit, Second Affiliated Hospital, Zhejiang University School of Medicine, Hangzhou 310051, Zhejiang Province, China.; 5 Department of Emergency, Wuxi People's Hospital affiliated to Nanjing Medical University, Jiangsu Province 214000, China.; 6 Lung Transplant Center, Wuxi People's Hospital affiliated to Nanjing Medical University, Jiangsu Province 214000, China.

**Keywords:** idiopathic pulmonary fibrosis, GZMK, TGF-β1, activin A

## Abstract

Idiopathic pulmonary fibrosis (IPF) is a fatal interstitial lung disease with progressive scarring and an unknown aetiology, but the cross-tissue immune network remains poorly understood. Here, we performed paired single-cell RNA sequencing and T cell receptor profiling of lung, mediastinal lymph node, and peripheral blood samples from patients with severe IPF. We identified a GZMK⁺CD8⁺ T cell subset enriched in fibrotic lung tissue with an inflammatory, low-cytotoxic phenotype, and a clonally related CD8_HSPA1A population enriched in lymph nodes, suggesting a cross-tissue clonal connection. Higher GZMK⁺CD8⁺ T cell signatures were associated with worse survival and impaired lung function. Functionally, GZMK overexpressing CD8⁺ T cells promoted fibroblast-to-myofibroblast differentiation and proliferation through TGF-β1/Activin A signalling, whereas GZMK knockdown attenuated these responses. Importantly, genetic ablation of GZMK markedly reduced collagen deposition and lung fibrosis in bleomycin-induced mice. Furthermore, inhibition of TGFβR1/ALK4 using the inhibitor TEW-7197 effectively suppressed fibroblast activation in vitro and significantly attenuated pulmonary fibrosis in bleomycin-induced mice. Together, these findings suggest a potential profibrotic immune-stromal circuit in severe IPF in which GZMK⁺CD8⁺ T cells promote fibroblast activation through TGF-β1/Activin A signalling, highlighting this pathway as a potential therapeutic target.

## Introduction

Idiopathic pulmonary fibrosis (IPF) is a progressive, fatal interstitial lung disease of unknown aetiology characterized by relentless fibrotic remodelling of the pulmonary parenchyma, ultimately culminating in end-stage respiratory failure 1. The median survival following diagnosis is approximately 2-4 years, with a 5-year survival rate less than 20%, underscoring the urgent unmet clinical need for novel therapeutic approaches 2,3. Histologically, the hallmark features of IPF include the aberrant proliferation and activation of fibroblasts, their transition into α-smooth muscle actin (α-SMA)-expressing myofibroblasts, and excessive extracellular matrix (ECM) deposition, culminating in irreversible fibrotic lesions termed “fibroblastic foci” 4.

Recent studies have increasingly highlighted the critical role of immune regulation in IPF pathogenesis 5. Mediastinal lymph node enlargement (MLNE), which is detectable by computed tomography (CT), has been reported in 40-80% of IPF patients 6,7. MLNE is correlated with higher total lung fibrosis scores, reduced diffusing capacity for carbon monoxide (DLCO), and worse prognosis compared to patients without lymphadenopathy, suggesting its potential utility as a predictive biomarker for disease progression 7-9. The precise biological mechanisms underlying MLNE in IPF remain undefined, but this phenomenon likely reflects heightened systemic immune activation that is integral to pulmonary pathology. In recent years, single-cell RNA sequencing (scRNA-seq) has significantly advanced our understanding of the pathogenesis of IPF 10,11. However, most studies have focused exclusively on lung tissue, and little is known about the relationship between immune cells in lymph nodes, peripheral blood, and the fibrotic lung microenvironment. Therefore, elucidating the distinctive lymphocyte characteristics and immune microenvironments across diverse tissue compartments in IPF with combined sequencing techniques is imperative for the development of effective therapeutic strategies.

Granzyme K (GZMK) is a serine protease secreted by lymphocytes that induces inflammatory cell death or promotes the release of proinflammatory cytokines through noncanonical pathways 12. In contrast to the classical cytotoxic molecule GZMB, GZMK is highly expressed in chronic inflammatory microenvironments and is proposed to function as an immunological mediator that both sustains cytotoxic immune responses and amplifies inflammatory signalling cascades 13. Recent studies in the contexts of cancer and chronic diseases have demonstrated that GZMK⁺CD8⁺ T cells exhibit distinct chemotactic and cytokine secretion profiles 14,15. Their activation can be directly induced by cytokines, providing a mechanistic rationale for their involvement in the pathogenesis of fibrosis.

Transforming growth factor-β (TGF-β) superfamily signalling is a central driver of fibrotic remodelling across multiple organs, including the lung. Among its members, TGF-β1 is widely recognized as a master regulator of fibrosis that promotes fibroblast activation, myofibroblast differentiation, and extracellular matrix deposition 16. TGF-β1 exerts its effects primarily through activation of the type I receptor TGFBR1 (also known as ALK5), thereby activating downstream Smad2/3 signalling 17. In IPF, elevated TGF-β1 signalling has been consistently observed and is considered a key mediator of fibroblast-to-myofibroblast transition and progressive tissue scarring 18. In addition to TGF-β1, Activin A, encoded by INHBA, has emerged as another important TGF-β superfamily ligand with roles in both immune regulation and tissue fibrosis 19. Activin A signals primarily through the type I receptor ALK4 (ACVR1B) to activate Smad2/3 signalling, and has been implicated in inflammatory responses, vascular remodelling, and fibrotic processes in multiple disease contexts 20. Notably, recent studies have demonstrated that Activin A can modulate T cell differentiation and function, suggesting that it may serve as a critical link between immune activation and stromal remodelling 21,22. However, the contribution of Activin A signalling in immune-stromal crosstalk, particularly in the context of CD8⁺ T cells and fibroblast activation in IPF, remains poorly defined.

In this study, we performed scRNA-seq on paired lung parenchyma, peripheral blood, and mediastinal lymph node samples from IPF patients. We identified a GZMK⁺CD8⁺ T cell subset that was selectively enriched in fibrotic lung tissue and characterized by a distinct inflammatory profile with relatively low cytotoxic potential. T cell receptor (TCR) sequencing further revealed a lymph-node-enriched CD8⁺ T cell population (CD8_HSPA1A), defined by high expression of the stress-response chaperone HSPA1A and transcriptional features of effector memory cells, which may represent a transitional state clonally linked to pathogenic CD8_GZMK cells detected in lung tissues. Through in vivo and in vitro experiments, we found that GZMK⁺CD8⁺ T cells can promote fibroblast-to-myofibroblast transition via the TGF-β1/Activin A signalling axis. Pharmacological or genetic disruption of this pathway attenuated pulmonary fibrosis, suggesting a previously unrecognized immune-stromal circuit contributing to IPF progression.

## Materials and Methods

### Ethics approval

This study was performed in accordance with the Declaration of Helsinki and approved by the Ethics Committee of The Second Affiliated Hospital Zhejiang University School of Medicine (ID: 2024-1416). All participants provided the written informed consent before inclusion. All animal study protocols were reviewed and approved by the Animal Care & Use Committee of The Second Affiliated Hospital Zhejiang University School of Medicine (ID: 2024-324).

### Study population and specimen collection

4 patients pathologically diagnosed with IPF who underwent lung transplantation at The Second Affiliated Hospital Zhejiang University School of Medicine were enrolled in this study. Inclusion criteria were: (i) a multidisciplinary and histopathological diagnosis of IPF according to international ATS/ERS guidelines; (ii) eligibility for lung transplantation; (iii) lymph node enlargement defined as a short-axis diameter ≥10 mm; and (iv) age≥18 years with the ability to provide written informed consent. Major exclusion criteria were: (i) evidence of connective tissue disease-associated ILD or other defined forms of interstitial lung disease; (ii) active pulmonary infection or malignancy at the time of transplantation; and (iii) prior lung transplantation. For each patient, peripheral blood was collected immediately before surgery, and matched fresh lung tissue and subcarinal mediastinal lymph nodes were obtained intraoperatively immediately after surgical resection. All specimens were promptly processed for single-cell analysis. Clinical characteristics and concomitant medications of the study participants are detailed in Supplementary Table 1. For validation studies using immunofluorescence staining, we conducted the staining procedure on IPF lung and lymph node specimens from the surgical cohort, along with control tissue from unused donor lungs.

Additionally, 12 patients with diagnosed IPF were enrolled for Spearman correlation analysis between GZMK expression levels in lung tissues and pulmonary function parameters. We further conducted co-culture cell interaction assay on 3 blood samples and 2 lung samples from IPF patients.

### Sample processing and scRNA sequencing

Fresh tissue specimens (lung and lymph nodes) were processed immediately after collection. Samples were washed in phosphate-buffered saline (PBS; Sangon) to remove blood, adipose tissue, and surface contaminants, then minced into approximately 0.5 mm^3^ fragments in RPMI-1640 medium (GNM11835-500, GENOM) supplemented with 5% Fetal Bovine Serum (FBS). Tissue digestion was performed at 37°C for 20 minutes using an enzymatic cocktail containing 0.35% collagenase type IV (Sigma, C5138-1G), 2 mg/mL papain (Sangon, A003124-0100), and 120 Units/mL DNase I (Sangon, A510099-0001). The resulting cell suspension was filtered through 70 μm strainers and centrifuged at 300 × g for 5 minutes at 4°C. Erythrocytes were lysed using erythrocyte lysis solution (130-094-183, Miltenyi) for 5 minutes at room temperature. Dead cells were removed using Dead Cell Removal MicroBeads (130-090-101, Miltenyi) according to the manufacturer's protocol. The final cell pellet was resuspended in PBS containing 0.04% BSA (Sigma, A1933-100G). From peripheral blood samples, peripheral blood mononuclear cells (PBMCs) were isolated within 2 hours of collection using Histopaque-1077 density gradient centrifugation (Sigma) following manufacturer's guidelines. For all samples, single-cell suspensions were adjusted to a final concentration of 700-1,200 cells/μL. Single-cell gene expression profiling and immune repertoire analysis were performed using the Chromium NextGEM Single Cell 5' Reagent Kits v2 (10x Genomics) according to standard protocols. Libraries were sequenced on an Illumina NovaSeq 6000 platform to achieve a minimum depth of 20,000 reads per cell.

### Data processing, quality control, normalization and clustering

Raw sequencing data were processed using the 10x Genomics Cell Ranger pipeline (v7.2.0). Reads were aligned to the human reference genome (GRCh38) and quantified to generate gene-cell count matrices. We implemented stringent quality control filters to remove low-quality cells. Specifically, cells were excluded if they expressed fewer than 200 unique genes or if their mitochondrial content exceeded 25% of total unique molecular identifier (UMI) counts. Potential cell doublets were computationally identified within each individual sample using DoubletFinder (v2.0.3) 23. Gene expression data were processed using Seurat (v5.1.0). Raw counts were normalized using the “NormalizeData” function, and the top 2,000 highly variable genes were identified for downstream analyses. Data were scaled using the “ScaleData” function, followed by principal component analysis (PCA) using 30 components. To correct for batch effects across tissue types (lung tissue, lymph nodes, and peripheral blood), we employed Harmony (v1.2.0) for data integration 24. Unsupervised graph-based clustering was performed using Seurat's “FindClusters” function across multiple resolution parameters (0.5-1.2). Cluster results were visualized using Uniform Manifold Approximation and Projection (UMAP). Differential gene expression analysis between clusters was conducted using the Wilcoxon rank sum test (logFC threshold > 0.25, adjusted P < 0.05). Cell type identities were assigned based on established marker genes from the CellMarker database and previous literature. For detailed analysis of specific cell populations, relevant clusters were subset into new Seurat objects using the “subset” function.

### Tissue distribution of clusters

To characterize the tissue distribution patterns of cellular populations, we quantified both the relative abundance and tissue-specific enrichment of each cell cluster. For relative abundance, we calculated the proportion of each cell type within the total cell population across samples. To determine tissue specificity, we computed an enrichment score using the ratio of observed to expected cell numbers (Ro/e) 25, following established methods. Expected cell numbers for each cluster-tissue combination were calculated using chi-squared test assumptions. Clusters with Ro/e values greater than 1 were considered enriched in the corresponding tissue type. This approach enabled systematic identification of tissue-preferential cell populations while controlling for differences in sampling depth across tissues.

### TCR analysis

The TCR sequences for individual T cells were acquired using the Cell Ranger toolkit (version 7.2.0) provided by 10x Genomics, employing the manufacturer-supplied human V(D)J reference genome “GRCh38-alts-ensembl”. Single-cell TCR sequences were matched to corresponding cellular transcriptomes using unique cell barcodes. Of the analyzed cells, 84.9% of T cells (32,125) yielded high-quality receptor sequences. Clonotypes were inferred using a CDR3 amino-acid-based definition, such that cells sharing identical productive CDR3 amino-acid sequences were assigned to the same clonotype. Expanded (clonal) T cells were defined as clonotypes observed in ≥2 cells, whereas singleton clonotypes (observed in only one cell) were classified as non-expanded (non-clonal). Cells lacking productive TCR contigs were excluded from clonotype-based analyses. Clonal overlap between different CD8⁺ T-cell subsets was quantified by computing a Morisita-Horn similarity index (0-1). The similarity matrix was visualized as a heatmap. We employed STARTRAC v0.1.0 to quantify three key metrics of T cell dynamics: clonal expansion (STARTRAC-expa), tissue migration (STARTRAC-migr), and state transition (STARTRAC-tran) 25.

To investigate the clonal relationship between lung GZMK⁺CD8⁺ and lymph node HSPA1A⁺CD8⁺ T cells, we performed comprehensive TCR repertoire analyses in Python using the scirpy, pandas, NumPy, scikit-learn, and SciPy packages. We calculated clonotype-level overlap (Jaccard similarity) and cell-level overlap (proportion of cells belonging to shared clonotypes) between the two populations. Statistical significance was assessed using Fisher's exact test. To evaluate whether TCRs from the two populations recognize similar antigens, we performed sequence-based clustering analysis on expanded clonotypes. Pairwise Levenshtein distances were calculated between all TCR CDR3 amino acid sequences, and dimensionality reduction was performed using t-distributed stochastic neighbor embedding (t-SNE, perplexity=30, 1000 iterations). To quantify TCR mixing, we calculated a mixing score for each TCR as the proportion of its k=10 nearest neighbors (by Levenshtein distance) belonging to the opposite tissue type. Statistical significance was assessed by permutation testing (1000 permutations) comparing observed mixing scores to null distributions generated by randomly shuffling tissue labels.

### Cell developmental trajectory analysis

To elucidate developmental relationships between cell populations, we employed Monocle2 26, and partition-based graph abstraction (PAGA) 27 to infer the lineage trajectories of the different cell subsets. Cell ordering along pseudo-temporal trajectories was inferred using Monocle2 (v2.30.1), with cells arranged in a branched structure based on transcriptional similarities. PAGA analysis was implemented through the Scanpy package to map the abstract graph topology of cellular transitions while preserving the global structure of the data.

### Functional pathway analysis

To characterize cell type-specific functional states, we curated published gene signatures associated with key biological processes. Pathway enrichment scores were calculated for individual cells using the AddModuleScore function in Seurat (v5.1.0) and visualized using UMAP dimensional reduction.

### Differential expression and pathway analysis

To identify cell type-specific markers, we performed differential expression analysis using Seurat's “FindAllMarkers” function with Wilcoxon rank-sum tests. Genes with adjusted *P* < 0.05 were considered significant markers for each cluster. For functional annotation, we conducted Gene Ontology (GO) and Kyoto Encyclopedia of Genes and Genomes (KEGG) pathway enrichment analyses using clusterProfiler (v4.12.6) 28.

### Cell-cell interaction analysis

To infer intercellular communication networks, cell-cell communication analysis was performed using CellPhoneDB (v5.0.1) with the v5.0.0 database. Normalized gene expression matrices and annotated cell identities were used as input. For each pairwise combination of cell types, statistically significant ligand-receptor interactions were inferred based on the expression of ligands in one cell type and corresponding receptors in another. Significance was assessed via permutation testing (n = 1000, *P* < 0.05). The number and strength of predicted interactions were visualized using circle plots. For key signalling pathways, selected ligand-receptor pairs were highlighted to illustrate specific intercellular communication patterns.

### Gene regulatory network analysis

To identify active transcription factors (TFs) and their downstream targets within fibroblast subpopulations, we implemented SCENIC (Single-Cell rEgulatory Network Inference and Clustering) v1.3.1 29. The analysis pipeline consisted of three main steps: First, we used GRNBoost to construct co-expression networks between TFs and their putative target genes from the raw count matrix. Next, we employed RcisTarget to identify direct regulatory relationships by evaluating the enrichment of TF binding motifs in the regulatory regions of co-expressed genes, resulting in high-confidence regulons. Finally, we quantified regulon activity scores for individual cells using AUCell and visualized the results to reveal cluster-specific regulatory programs. This approach enabled systematic identification of master regulators driving fibroblast heterogeneity in IPF.

### Validation using public RNA sequencing datasets

To validate single-cell findings at the transcriptomic level, we analyzed two bulk transcriptomic datasets, GSE28042 and GSE213001, which included 72 IPF samples, and 61 IPF samples and 76 control samples, respectively. The GSVA package (v1.52.3) was employed to calculate cellular subtype enrichment profiles across individuals, utilizing a gene signature set comprising the top 50 differentially expressed markers for each defined cell cluster. Survival outcomes in the GSE28042 dataset were assessed using multivariable Cox proportional hazards regression. Patients were stratified into high- and low-score groups using the median CD8_GZMK signature score as the cutoff. Kaplan-Meier survival curves and time-dependent receiver operating characteristic (ROC) analyses were generated using the R survival package (v3.7.0). The GSE213001 dataset was used for transcriptomic correlation analysis and to assess the infiltration levels of specific T cell subsets. The Pearson correlation between the abundance of GZMK⁺T cells and CTHRC1⁺fibroblasts was calculated, and a cell type-level correlation matrix was generated based on enrichment scores across individuals.

Public scRNA-seq dataset GSE214085, comprising 10 IPF lung samples and 8 normal lung samples, was analyzed to validate the reproducibility of our single-cell findings. The dataset was processed using a workflow consistent with that used for our cohort, and cell-type proportions and selected gene signature scores were compared at the individual sample level where applicable. In addition, scRNA-seq data from IPF lymph nodes in our cohort were integrated with normal lymph node data from GSE131907, which included 10 normal lymph node samples. After batch-effect correction using Harmony and unified clustering, CD8⁺T cells were extracted, and the CD8_HSPA1A signature score was calculated using AddModuleScore to assess stress-related CD8⁺T cell alterations. All statistical analyses comparing cell-type proportions were conducted strictly at the patient level.

### GZMK recombinant plasmid construction

According to the nucleotide sequence of human GZMK (NM_002104) from NCBI databases. We constructed a recombinant lentivirus plasmid (pcDNA3.1-Cppt-IRES) with full length nucleotide sequence of GZMK (795 bp, from 5' to 3'). The catalytically inactive GZMK-S214A mutant was generated by PCR-based site-directed mutagenesis 30. The GZMK full-length plasmids were purchased from Vigene Bioscience. In preparation for lentiviral supernatant, before transduction, HEK293T cells were seeded at 6 × 10^6^ cells per 100 mm dish with 80% cell confluency. The lentiviruses were packaged by co-transfecting HEK293T cells with a lentivirus plasmid containing GZMK gene sequence or GZMK-shRNA plasmid, encoding the VSV-G envelope protein plasmid, and the packaging plasmid psPAX2, using PEI transfection mediator. The fresh medium was added after 8 hours. After 48 h, the lentivirus supernatants were all collected and filtered using a 0.45 μm membrane to remove cell debris. Then the lentiviruses were concentrated by ultracentrifugation (Optima XPN-100, Beckmann) at 82,700 × g for 2 h at 4 °C.

### Isolation and culture of human CD8^+^T cells

PBMCs were obtained from healthy donors and IPF patients by Ficoll-Hypaque (TBD Science) density-gradient centrifugation. Cell number and viability were assessed by trypan blue exclusion, and only PBMC preparations with viability >90% were used for downstream experiments. CD8⁺ T cells were negatively isolated from PBMCs using the CD8⁺ T-lymphocyte enrichment set DM (BD-IMag™, 557941) according to the manufacturer's instructions, and then activated with 10 ng/mL recombinant human IL-2 (eBioscience), 1 μg/mL anti-CD3 (eBioscience) and 1 μg/mL anti-CD28 (eBioscience) antibodies for 48 h. After lentiviral transduction, CD8⁺ T cells were cultured and expanded in RPMI 1640 (Gibco) supplemented with 10% FBS (Gibco) and 1% penicillin-streptomycin solution (Yeasen), in the continued presence of 10 ng/mL IL-2, 1 μg/mL anti-CD3 and 1 μg/mL anti-CD28 until use in co-culture assays.

### CD8^+^T cells and human fetal lung fibroblasts co-culture assay

In vitro non-contact co-culture assays were used to study the interaction between CD8⁺ T cells and human fetal lung fibroblasts (HFL1). HFL1 cells were maintained in F-12K Nutrient Mixture (Gibco, 21127022) supplemented with 10% FBS (Gibco) and 1% penicillin-streptomycin and were used at passages 2-5. An amount of 1 × 10⁵ HFL1 cells per well was seeded into the lower chamber of a 24-well cell culture plate and allowed to adhere overnight. Activated CD8⁺ T cells were cultured in RPMI 1640 (Gibco) with 10% FBS (Gibco) and 1% penicillin-streptomycin, and 1 × 10⁵ CD8⁺ T cells per well were seeded into 0.4-μm pore-size transwell inserts with 24-well plates (Corning, 3450) placed above the HFL1 monolayer. A 0.4-μm pore size was chosen to prevent cellular migration between compartments while allowing the diffusion of soluble factors. After 40 h of co-culture, CD8⁺ T cells were removed and fibroblasts were collected for subsequent experiments.

### PBMC and HFL1 co-culture assay

For PBMC-fibroblast co-culture experiments, HFL1 cells were seeded and maintained in the lower chamber as described above. Freshly isolated PBMCs were resuspended in RPMI 1640 (Gibco) supplemented with 10% FBS (Gibco) and 1% penicillin-streptomycin and pre-stimulated with recombinant human IL-2 (10 ng/mL). PBMC number and viability/proliferative capacity were evaluated by BrdU incorporation using flow cytometry, and only preparations with adequate viability were used for co-culture. 5 × 10^5^ PBMCs were then added to 0.4-μm pore-size transwell inserts (24-well format, Corning) placed above the HFL1 monolayer. After 4 h of non-contact co-culture, bosutinib (10 μM) or TEW-7197 (10 μM) was added to the upper chambers, and the cultures were maintained for an additional 36 h. At the end of co-culture, HFL1 cells and/or PBMCs and supernatants were collected for subsequent analyses as indicated.

### CD8^+^T cells of IPF patients and HFL1 co-culture assay

After CD8^+^T cells isolated from PBMCs of IPF patients, CD8⁺ T cells were cultured in RPMI 1640 (Gibco) added with 10% FBS (Gibco) and 1% penicillin-streptomycin activated with 10 ng/mL IL-2, 1 μg/mL anti-CD3 and 1 μg/mL anti-CD28, treated with or without 100 ng/mL Activin A-neutralizing antibody (Universal Biotech) and 5 μg/mL TGFβ1-neutralizing antibody (proteintech) for 2 days. For CD8^+^T cells of IPF patients-HFL1 co-culture assay, HFL1 were cultured at passage 2-5 and were seeded into the 24-well cell plate (Corning) at an amount of 1 × 10^5^ HFL1 per well. An amount of 1 × 10^5^ CD8^+^ T cells of IPF patients was seeded into 0.4 μm transwell-clear inserts with 24-well cell plate (Corning). After co-culture for 40 hours, CD8^+^ T cells were removed and HFL1 were collected for further experiments.

### Primary lung fibroblasts of IPF patients' isolation and culture

Lung tissue blocks were harvested under a sterile laminar flow hood. The blocks were repeatedly washed three times with PBS containing 1% penicillin-streptomycin. Using sterile scissors, the tissue was minced as small as possible to approximately 1.5-2 mm^3^. The minced tissue was dissociated and the suspension was loaded into a 15 mL centrifuge tube and was centrifuged at 1500 rpm for 15 minutes. The supernatant was discarded, and the pellet was resuspended in Dulbecco's Modified Eagle Medium (DMEM) supplemented with 10% FBS and 1% penicillin-streptomycin. The suspension was thoroughly pipetted up and down to achieve uniform dispersion. The minced tissues were seeded in a T25 culture flask, evenly spread to cover the bottom, and 2 mL of DMEM with 10% FBS and 1% penicillin-streptomycin was added. The flask was incubated at 37°C with 5% CO_2_ for 1 hour to facilitate initial attachment. After 1 hour, the flask was inverted and incubated for an additional 24 hours. 5 mL of complete culture medium, DMEM with 10% FBS and 1% antibiotic-antimycotic, was added with the flask in the upright position. Medium was changed every 3-4 days. Primary fibroblasts were used for next experiments at passages 2-5.

### Transfection of ALK4-shRNA and ALK5-shRNA plasmid into primary lung fibroblasts of IPF patients

Using passage 2-5 lung fibroblasts isolated from IPF patients, before transfection, cells that are 75-85% confluent in a 60-mm cell dishes are dissociated. After counting the cells using trypan blue exclusion, about 1 × 10^5^ cells in 500 µL growth medium were seeded into a single well of a 24-well cell plate. On the day of transfection, ALK4-shRNA (Vigene Bioscience) and ALK5-shRNA (Vigene Bioscience) plasmids were transfected into fibroblasts using Lipofectamine 3000 Transfection Reagent (Thermo) according to the manufacturer's instructions.

### Proliferation assay and flow cytometry

Flow cytometry was used to assess cellular proliferation by measuring Ki-67 expression and BrdU incorporation. PBMCs were separated from peripheral blood of healthy controls and IPF patients for flow cytometry analysis by density gradient centrifugation using Ficoll-Hypaque (TBD science, LDS1075). 10 µM BrdU (Yeasen, 40204ES80) were added in HFL1 cells and incubated for 2 h. HFL1 cells and PBMCs were washed by PBS containing 2% FBS and then fixed by 4% PFA. For intracellular cytokine, Ki67 and BrdU staining, 1 × 10^6^ cells were permeabilized with Permeabilization Wash Buffer (Yeasen, 40403ES64) and stained with fluorescently-conjugated antibodies for 30 mins at room temperature according to manufacturer's protocols. For surface staining, 1 × 10^6^ cells were stained with fluorescently-conjugated antibodies for 30 mins at room temperature. Staining antibodies for flow cytometry analysis include FITC-anti-CD3 (Thermofisher, 11-0037-41), PE-anti-CD8 (Thermofisher, 12-0088-42), anti-GZMK (Abcam, ab288726), IgG H&L (Alexa Fluor**®** 647, Abcam, ab150075), Alexa Fluor™ 488-anti-Ki67 (Thermofisher, 53-5698-80) and FITC-anti-BrdU (Thermofisher, 11-5071-41). After washed with PBS, stained cells were recorded on Cytoflex (Beckman Coulter) and further analyzed by FlowJo_V10 software.

### ELISA assay

The levels of GZMK in serum of bleomycin-induced IPF mice were analyzed using mouse GZMK (Hengrui Hongchuang Technology Development Co., China) ELISA kits according to the manufacturer's instructions. The absorbance was read at 450 nm, and the results are presented as nanogram (ng) per liter (L).

### RNA extraction and quantitative real-time PCR

Total RNA was extracted from cultured cells or tissue samples using the RNAiso Plus reagent (Takara, 9108) according to the manufacturer's instructions. The concentration and purity of RNA were determined using a NanoDrop spectrophotometer (Thermo Scientific). Complementary DNA (cDNA) was synthesized using a PrimeScript™ RT reagent Kit with gDNA Eraser (Takara, RR047A) in a total volume of 20 µL. Quantitative real-time PCR (qRT-PCR) was performed using TB Green® Premix Ex Taq™ II (Takara, RR820A) on a LightCycler® 480 System (Roche). Gene expression levels were normalized to GAPDH and calculated using the 2^^-ΔΔCt^ method.

### Western blotting

Cells or tissue samples were lysed in RIPA buffer (Beyotime, P0013B) supplemented with protease and phosphatase inhibitor cocktails (Roche). Protein concentration was determined using the BCA Protein Assay Kit (Thermo Fisher). Equal amounts of protein (20-40 μg) were resolved by SDS-PAGE (10-12% gels) and transferred onto PVDF membranes (Millipore). Membranes were blocked with 5% non-fat milk in TBST (Tris-buffered saline with 0.1% Tween-20) for 1 hour at room temperature, followed by overnight incubation at 4°C with primary antibodies. After washing, membranes were incubated with HRP-conjugated secondary antibodies for 1 hour at room temperature. Signals were detected using ECL Western blotting substrate (Bio-Rad) and visualized using a ChemiDoc Imaging System (Bio-Rad). Densitometric analysis was performed using ImageJ software. Primary antibodies include anti-GZMK (CSB, PA002797), anti-α-SMA (Proteintech, 67735-1-Ig), anti-collagen I (Abcam, ab260043), anti-pSmad2 (Abclonal, AP0548) and anti-Smad2 (Abclonal, A7699).

### Hematoxylin and Eosin (H&E) staining

Lung tissues were fixed in 4% paraformaldehyde for 48 hours, embedded in paraffin, and sectioned at 3 μm thickness. Sections were deparaffinized, rehydrated, and stained with hematoxylin for 5 minutes, followed by eosin staining for 2 minutes. After dehydration in graded ethanol and clearing in xylene, slides were mounted with neutral resin and examined under a light microscope (Olympus).

### Masson's trichrome staining

Paraffin-embedded tissue sections were deparaffinized, rehydrated, and subjected to Bouin's fixative at 56°C for 1 hour. Sections were stained sequentially with Weigert's iron hematoxylin, Biebrich scarlet-acid fuchsin, phosphomolybdic-phosphotungstic acid, and aniline blue. Slides were dehydrated, cleared in xylene, and mounted. Collagen fibers stained blue, nuclei black, and cytoplasm red. Fibrosis severity was semi-quantitatively evaluated using the Ashcroft scoring system by two independent blinded observers.

### Immunohistochemistry (IHC)

Paraffin-embedded lung tissue sections were deparaffinized and rehydrated. Antigen retrieval was performed using sodium citrate buffer (pH 6.0) in a microwave for 24 minutes. Endogenous peroxidase activity was quenched with 3% hydrogen peroxide for 10 minutes. Sections were blocked with 5% normal goat serum for 30 minutes at room temperature, then incubated with primary antibodies overnight at 4°C. After PBS washes, sections were incubated with HRP-conjugated secondary antibodies for 30 minutes at room temperature. DAB substrate was used for color development, and nuclei were counterstained with hematoxylin. Slides were dehydrated, mounted, and imaged using a brightfield microscope. The following primary antibodies were used: anti-GZMK (CSB, PA002797), anti-ALK4 (abclonal, A2279).

### Multiplexed immunofluorescence staining

Surgical specimens were immediately fixed in neutral buffered formalin for 48 hours, followed by standard dehydration and paraffin embedding procedures. Five-micrometer sections were cut from paraffin blocks and mounted on positively charged glass slides. Prior to staining, sections underwent deparaffinization by baking at 70°C for 1 hour, followed by xylene treatment and rehydration through a graded ethanol series (100%, 85%, and 75%). Antigen retrieval was performed using EDTA buffer (pH 9.0) under optimized microwave conditions. To minimize non-specific binding, sections were blocked with 10% normal goat serum for 30 minutes at room temperature. Primary antibodies were applied and incubated overnight at 4°C in a humidified chamber. The following primary antibodies were used: anti-CD8 (Abcam, ab237709), anti-GZMK (Cell Signaling Technology, 98946), and anti-HSP70 (Proteintech, 10995-1-AP). Following PBS washes, sections were incubated with horseradish peroxidase-conjugated secondary antibodies according to manufacturers' recommendations. Nuclear counterstaining was performed using DAPI.

### Bleomycin-induced pulmonary fibrosis mouse model

To induce pulmonary fibrosis, 6-8-week-old male C57BL/6 mice (20-25 g) were anesthetized and administered bleomycin sulfate (2.5 mg/kg body weight) dissolved in sterile PBS via intratracheal instillation. Control mice received an equal volume of sterile PBS. Mice were monitored daily for signs of distress or weight loss. Tissues and blood were harvested at day 21 post-BLM for histological and molecular analysis. In selected experiments, bosutinib (3.3 or 6.6 mg/kg) was administered to inhibit GZMK activity; TEW (vactosertib, TEW-7197), a dual TGFβR1/ALK4 inhibitor, was used to block downstream signalling; and FTY720 was used to promote lymphocyte retention in lymph nodes and limit egress to peripheral tissues. Lungs and blood were collected at day 21 for histological, molecular and biochemical analyses. GZMK KO mice are provided by Cyagen Biosciences.

### Statistical analysis

All statistical analyses were performed using GraphPad Prism (version 9.5.0) and R (version 4.4.0). For comparisons between two groups on cell-level, statistical significance was determined using either two-tailed Student's t-tests (for normally distributed data) or Wilcoxon rank-sum tests (for non-parametric data). Correlation between continuous variables were evaluated using Spearman's rank correlation coefficient (r_s_). For multiple group comparisons on cell-level and patient-level, one-way ANOVA was performed followed by Tukey's post-hoc test to correct for multiple testing. Statistical significance was defined as P < 0.05 for all analyses. Detailed statistical methods for specific comparisons are provided in the corresponding figure legends. Gemini (Google LLC) was used exclusively for limited visual refinement of a schematic figure.

## Results

### Single-cell landscape of cell composition in lung tissues, lymph nodes, and peripheral blood in IPF patients

To comprehensively characterize the immune microenvironment in IPF, we performed scRNA-seq and TCR repertoire profiling on 11 samples from four IPF patients. These samples included matched pulmonary tissues (PT), mediastinal lymph nodes (LN), and peripheral blood (PB) (Figure 1A). After stringent quality control filtering, doublet exclusion, and normalization, a total of 83,319 high-quality single cells were retained for downstream analysis.

Unsupervised clustering and t-distributed stochastic neighbour embedding (t-SNE) visualization revealed nine major cell populations based on classical marker gene expression, including T cells, B cells, natural killer (NK) cells, myeloid cells, epithelial cells, endothelial cells, fibroblasts, mast cells, and platelets (Figure 1B, Figure S1A). t-SNE plots stratified by sample origin including PT, LN, and PB revealed distinct tissue-specific clustering patterns (Figure 1C). The marker genes defining each cluster are illustrated in Figure 1D (see also Figure S1C).

To elucidate the cellular composition across the PT, LN, and PB samples of IPF patients, we analysed the tissue-specific distributions of cell types (Figure 1E-F, Figure S1B). The LN samples were enriched in T and B lymphocytes, which is consistent with their immune surveillance function. PB samples contained relatively high proportions of T and NK cells. PT samples showed prominent stromal cell components and immune cell infiltration characterized by a substantial proportion of T cells and myeloid cells, highlighting the immunologically active microenvironment within IPF lung tissues. Consequently, we focused on these immune cell subsets to explore their roles in IPF progression and differentiation across tissue compartments.

### Proinflammatory GZMK^+^CD8^+^ T cells are enriched in IPF lung tissues

Through unsupervised clustering analysis of 40,635 cells across all samples, we identified five CD4^+^ T cell clusters, four CD8^+^ T cell clusters, one NKT cell cluster, and one MAIT cell cluster (Figure 2A, Figure S2A-C). Tissue distribution analysis revealed compartment-specific enrichment of T cell subsets, with notable differences in CD8⁺ T cell composition across tissues (Figure 2B).

Among these, CD8_CCR7 cells represent naïve T cells with high *CCR7*, *SELL*, and *TCF7* expression. CD8_CX3CR1 cells showed high expression of *CX3CR1* and *KLRG1* together with a terminal cytotoxic program (*PRF1/GZMB/NKG7/GNLY/FGFBP2*), consistent with terminally differentiated cytotoxic effector-memory (Temra) cells, whereas the CD8_HSPA1A and CD8_GZMK subsets presented transcriptional signatures of effector memory (Tem) cells with distinct functional features (Figure 2C, Figure S2B). Notably, CD8_HSPA1A exhibited higher expression of heat shock protein family genes. Proportional analysis revealed that the CD8_GZMK subset was markedly enriched in PT samples, while CD8_CX3CR1 cells were more abundant in PB samples, and CD8_HSPA1A cells preferentially localized to LN samples (Figure 2D, Figure S2D). Among CD8⁺ T cell subsets, CD8_CX3CR1 cells showed the highest cytotoxicity scores, whereas CD8_GZMK cells had lower cytotoxicity scores than CD8_CX3CR1 cells, together with the higher inflammatory and exhaustion scores (Figure 2E, Figure S2E-F).

To explore the functional roles of distinct CD8⁺ T cell subsets in IPF, we performed pathway enrichment analysis on the differentially expressed genes in each cluster (Figure 2F). The CD8_GZMK population, enriched in fibrotic lung tissues, showed significant upregulation of pro-inflammatory pathways including TNF signalling, chemokine signalling, and T cell receptor signalling. In contrast, CD8_CX3CR1 cells were enriched for cytotoxic immune response pathways, while CD8_HSPA1A cells exhibited upregulation of antigen presentation and immune modulation pathways. Furthermore, regulon analysis using SCENIC revealed that CD8_GZMK cells were transcriptionally driven by proinflammatory and immediate-early transcription factors, including *JUN*, *JUND*, *BCL3*, and *EGR1* (Figure 2G). These TFs are known to regulate T cell activation, cytokine production, and tissue remodelling. To further explore transcriptional regulation, we constructed a TF-target gene interaction network (Figure 2H), identifying *JUN*, *JUND*, *BCL3*, and *EGR1* as central hub regulators connected to fibrosis- and inflammation-associated genes such as *PTGER4*, *NR4A2*, *CD44*, and *FOS*. This network suggests that CD8_GZMK cells may sustain chronic immune activation via a tightly coordinated regulatory program.

We further examined the expression of chemokines, chemokine receptors, and inflammatory cytokines across clusters (Figure 2I-J). CD8_GZMK cells exhibited upregulated expression of several chemokine receptors, including *CXCR4*, *CCR4*, and *CXCR6*, as well as chemokines such as *CCL3L3*, facilitating immune cell recruitment to inflamed tissues (Figure 2I). Additionally, CD8_GZMK cells exhibited marked upregulation of proinflammatory cytokines such as *IL17A*, *IL26*, and *IL1B* (Figure 2J). Notably, unlike the CD8_CX3CR1 subset, which showed high expression of cytotoxic genes, CD8_GZMK cells exhibited a hybrid phenotype characterized by inflammatory cytokine production with limited cytotoxic potential.

To examine whether our findings could be reproduced in an independent cohort, we analyzed the publicly available scRNA-seq dataset GSE214085, which includes samples from patients with 10 IPF and 8 healthy controls (Figure S3A-B). Cell-type annotation showed high prediction confidence, as reflected by high prediction scores for CD8_GZMK in the independent dataset (Figure S3C). Analysis of cell-type proportions revealed a higher relative abundance of CD8_GZMK in IPF samples (Figure S3D). Consistent with our primary dataset, quantitative analysis confirmed that the proportion of CD8_GZMK cells was significantly increased in IPF compared with healthy controls (Figure S3E; *P* < 0.05). Together, these results characterize CD8_GZMK cells as a low-cytotoxicity, inflammation-skewed effector memory CD8⁺ T cell population enriched in the fibrotic lung, which may contribute to persistent immune activation and immune-mediated fibrogenesis in IPF.

Following our investigation into CD8⁺ T cell heterogeneity, we further examined the distribution and functional characteristics of CD4⁺ T cells in IPF (Figure S2A, Figure S2G). The CD4_CCR7 subset, which is characterized by the expression of classical naïve T-cell markers, was predominantly enriched in the LN and PB. The CD4_ANXA2 subset, characterized by elevated expression of *ANXA2*, *ANXA1*, and *ICAM2*, corresponded to central memory-like T cells and was broadly distributed across PB, LN, and PT. In contrast, CD4_CD69 cells expressing the tissue-resident marker *CD69*, CD4_FOXP3 cells expressing the regulatory marker *FOXP3*, and CD4_CXCR5 cells expressing T follicular helper markers (*CXCR5* and *BCL6*) were primarily localized to the LN and PT, with the greatest enrichment in the LN.

### TCR analysis revealed a close relationship between GZMK^+^CD8^+^ T cells and HSPA1A^+^CD8^+^ T cells

To investigate the clonal architecture and developmental trajectories of T cell subsets in IPF, we performed TCR sequencing and successfully recovered paired TCR α and β chains in 32,125 T cells across all samples. Notably, compared with CD4⁺ T cells, CD8⁺ T cells exhibited a substantially greater degree of clonal expansion, suggesting that antigen-driven proliferation occurred within the fibrotic lung microenvironment (Figure 3A-B). Among tissues, T cells from PT samples showed the highest proportion of expanded clones, in contrast to the more quiescent profiles observed in PB and LN samples, indicating robust local immune activation at the site of fibrosis. Further analysis of T cell clonal expansion in IPF revealed prominent clonal amplification within the CD8_CX3CR1, CD8_GZMK, and CD8_HSPA1A subsets, highlighting sustained or highly active immune responses within the local microenvironment (Figure 3C).

To further delineate tissue-specific clonal dynamics, we profiled the top 50 expanded clones in each compartment. In PT, the top expanded clones were overwhelmingly dominated by the CD8_GZMK subset, with minimal contributions from other T-cell subsets (Figure 3D). This striking enrichment suggests that CD8_GZMK cells are the predominant antigen-experienced population at the site of fibrosis and may directly contribute to local inflammation and tissue remodelling in IPF. In the LN, the clones were more broadly distributed across the CD8_CX3CR1, CD8_GZMK, and CD8_HSPA1A subsets, indicating active T cell priming and diverse antigenic experience within the lymphoid compartment (Figure 3E). In contrast, the top clones in the PB were largely confined to the CD8_CX3CR1 subset, reflecting a circulating cytotoxic effector population that may play a role in immune surveillance and systemic immune responses (Figure 3F).

To further characterize T cell behaviour, we applied the STARTRAC framework to quantify clonal expansion, migration, and state transition indices across CD8⁺ T cell subsets (Figure 3G). The CD8_GZMK and CD8_CX3CR1 subsets exhibited the highest expansion scores, reflecting their strong clonal proliferation in the tissue microenvironment. Interestingly, CD8_HSPA1A cells presented the highest migration and transition indices, suggesting that these cells, enriched in the LN, may possess migratory and plastic phenotypes. To explore the developmental relationships among CD8⁺ T cell populations, we performed a PAGA trajectory analysis, which revealed a close connection between the CD8_HSPA1A and CD8_GZMK subsets (Figure 3H). This relationship was further supported by the results of the clonal similarity analysis, in which CD8_HSPA1A and CD8_GZMK displayed the highest degree of clonal similarity among all the subsets (Figure 3I), highlighting a strong clonal relatedness between these two populations.

Integrated TCR analyses were performed to define the relationship between lymph node CD8_HSPA1A cells and lung CD8_GZMK cells. Clonotype analysis identified 78 shared TCR clonotypes, representing 8.5% of total clonotypes but accounting for 35.8% of lung CD8_GZMK cells and 45.4% of lymph node CD8_HSPA1A cells (Figure S4A). Shared clonotypes were predominantly highly expanded clones. The largest shared clonotype comprised 276 cells across the two tissues, indicating substantial clonal expansion likely driven by antigen recognition (Figure S4B-C). Analysis of TRBV and TRBJ gene usage further revealed highly similar patterns between the two populations (Figure S4D-E). Sequence-based TCR clustering further indicated substantial overlap and intermixing of lung- and lymph node-derived TCRs, rather than tissue-specific segregation (Figure S4F-G). These findings suggest that CD8_HSPA1A cells in the lymph nodes may represent a transitional state clonally linked to lung CD8_GZMK cells.

To further investigate CD8⁺ T cell alterations in lymph nodes, we integrated scRNA-seq data from IPF lymph nodes in our cohort with normal lymph node data obtained from the publicly available dataset GSE131907 (Figure S3F-G). Calculation of the CD8_HSPA1A gene signature score showed elevated expression of this stress-related program in a subset of CD8⁺ T cells, which was more prominent in IPF lymph nodes (Figure S3H). Quantitative analysis further showed that the proportion of CD8_HSPA1A cells was significantly increased in IPF lymph nodes compared with normal lymph nodes (Figure S3I; *P* = 0.024**)**. Multiplex immunofluorescence staining further confirmed a marked increase in CD8⁺HSP70⁺ cells within the lymph nodes of IPF patients compared with those of healthy controls, indicating an enhanced stress response and immune activation in IPF microenvironment (Figure 3J). These findings support the presence of disease-associated T cell activation or stress responses within the lymph nodes in IPF.

### GZMK⁺CD8⁺ T cells are increased in IPF and correlated with fibrosis severity and poor prognosis

To investigate the prognostic implications of this subset, we stratified patients from the publicly available GSE28042 cohort into high- and low-score groups using the median CD8_GZMK signature score as the predefined cutoff and conducted a Kaplan-Meier survival analysis. Compared with patients with lower scores, patients with higher CD8_GZMK scores had significantly shorter overall survival (*P* < 0.0001; Figure 4A), indicating that elevated numbers of GZMK⁺CD8⁺ T cells may serve as a predictor of poor clinical outcomes.

Histopathological assessment using haematoxylin and eosin (H&E) and Masson's trichrome staining of lung sections from IPF patients further revealed severe disruption of alveolar architecture and extensive collagen deposition in IPF lungs, consistent with progressive fibrotic remodelling (Figure S5A). Both qPCR and Western blotting revealed significantly elevated expression of fibrosis-related markers (COL1A1 and α-SMA) and GZMK in IPF lung tissue samples compared with control tissue samples (Figure 4B-C). Multiplex immunofluorescence staining revealed marked accumulation of GZMK⁺CD8⁺ T cells in fibrotic lung tissue but not in control lung tissue (Figure 4D), suggesting the selective enrichment of this subset within the fibrotic microenvironment.

To further elucidate the functional significance of GZMK expression in relation to disease severity, we examined the relationship between lung tissue GZMK levels and clinical pulmonary function parameters from twelve IPF patients, including forced vital capacity (FVC) and DLCO. Correlation analysis demonstrated that higher GZMK expression was associated with lower DLCO (% predicted) and reduced FVC (% predicted) (Figure 4E-F; Figure S5B), reinforcing its potential role in the pathogenesis of fibrosis and highlighting its utility as a prognostic biomarker.

### GZMK⁺CD8⁺ T cells-fibroblasts interaction drives fibrogenesis in IPF

We performed unsupervised clustering analysis on 2,659 high-quality fibroblasts and classified them into seven distinct subpopulations through manual annotation combined with established marker genes 31,32 (Figure 5A). These comprised four canonical fibroblast subtypes and three specialized subsets: smooth muscle cells, pericytes, and mesothelial cells (Figure 5B).

To evaluate the fibrotic potential of these fibroblast subsets, we analysed the expression of ECM-related genes and calculated fibrosis scores. The FIB_CTHRC1 population exhibited the highest expression of *ACTA2*, *FN1*, and multiple collagens, which is consistent with a robust myofibroblast phenotype (Figure S5C). Consistent with these findings, FIB_CTHRC1 had the highest fibrosis score among all the fibroblast types, followed by FIB_LGR5 and FIB_LIMCH1, whereas FIB_SFRP1 had the lowest fibrosis score (Figure 5C, Figure S5D). Monocle-based pseudotime trajectory analysis revealed a progressive trajectory of fibroblast differentiation, with FIB_SFRP1 localized at the origin and FIB_CTHRC1 at the terminal end, supporting a progressive activation process (Figure 5D, Figure S5E). Pathway enrichment analysis further revealed distinct functional profiles across fibroblast subsets (Figure 5E). FIB_LIMCH1 fibroblasts were enriched in pathways related to the mechanical stress response and cytoskeletal organization, such as PI3K-PKB signalling and the regulation of actin dynamics. FIB_LGR5 cells were enriched in mesenchymal proliferation and TGF-β signalling pathways, suggesting a profibrotic profile. The FIB_CTHRC1 subset was associated with extracellular matrix organization and collagen metabolic processes, reinforcing its central function in fibrotic remodelling. Conversely, FIB_SFRP1 fibroblasts were enriched in immune-related pathways, including antigen presentation, JAK-STAT signalling, and complement activation, indicating potential immunoregulatory roles.

To explore the crosstalk between fibroblasts and T cells, we performed ligand-receptor interaction analysis focusing on the TGF-β and CXCL signalling axes. The results revealed that CD8_GZMK cells may interact with the FIB_CTHRC1 and FIB_LGR5 subsets via the *TGFβ1-TGFBR1/TGFBR2* axis, suggesting that CD8_GZMK cells promote myofibroblast activation and progression of fibrosis through the TGFβ pathway (Figure 5F). In addition, FIB_CTHRC1 cells strongly interacted with CD8_GZMK cells via the *CXCL14-CXCR4* and *CXCL16-CXCR6* axes, suggesting that fibroblasts may actively recruit CD8_GZMK cells to fibrotic regions through chemokine secretion (Figure S5F). We next examined the expression of TGF-β superfamily ligands across CD8^+^ T cell subsets (Figure 5G). Notably, the expression of *TGFB1*, *TGFB2* and *INHBA* (encoding activin A) was elevated in CD8_GZMK cells, suggesting that these cells can directly contribute to TGF-β-mediated fibrotic signalling.

Finally, ssGSEA of the GSE213001 dataset revealed significantly higher infiltration scores of CD8_GZMK cells in IPF tissues than in normal control tissues (Figure S5G), indicating disease-associated enrichment of this subset. Correlation analysis of cell type enrichment scores revealed a strong positive correlation between CD8_GZMK cells and the FIB_CTHRC1 fibroblast subset (Figure 5H). This relationship was further validated by a significant Spearman correlation (R = 0.63) between the abundance of CD8_GZMK and FIB_CTHRC1 cells, further supporting a potential link between CD8_GZMK cells and fibroblast-mediated fibrogenesis (Figure 5I).

### GZMK overexpression in CD8⁺ T cells promotes fibroblast activation and proliferation via TGF-β signalling

To determine the direct functional impact of GZMK expression in CD8⁺ T cells on fibroblast function, we established an in vitro coculture model using HFL1 and human peripheral CD8⁺ T cells genetically modified to overexpress (GZMK-OE) or knockdown (GZMK-shRNA) GZMK (Figure 6A). Efficient GZMK modulation in CD8⁺ T cells was confirmed at both the mRNA and protein levels by qPCR and Western blotting, respectively (Figure 6B-C). Compared with vector controls, GZMK-OE CD8⁺ T cells exhibited enhanced secretion of Activin A and TGFβ1, along with increased mRNA expression of *INHBA* and *TGFB1*, whereas GZMK knockdown decreased both the protein secretion and gene expression of these cytokines (Figure 6D, Figure S6A). Functionally, coculture of fibroblasts with GZMK-OE CD8⁺ T cells markedly upregulated the protein expression of the fibrotic markers α-SMA and Collagen I whereas GZMK knockdown significantly suppressed the expression of these genes, indicating that high GZMK expression in CD8⁺ T cells promotes fibroblast activation (Figure 6E). In addition, direct stimulation of fibroblasts with recombinant GZMK also significantly upregulated the mRNA expression of α-SMA and COL1A1 in a dose-dependent manner (Figure S6B).

To assess whether this coculture-induced activation requires functional GZMK and activin activity, we introduced CD8⁺ T cells expressing a function-deficient GZMK mutant (Figure S6C) and pharmacologically blocked Activin A using follistatin, an activin-binding inhibitor. Fibroblasts cocultured with GZMK-OE CD8⁺ T cells exhibited a marked upregulation of* α-SMA* and *COL1A1* mRNA expression, which was markedly attenuated by either the GZMK mutant or follistatin (Figure 6F). Consistently, GZMK-OE CD8⁺ T cells significantly enhanced fibroblast proliferation, as measured by flow cytometric analysis of the percentages of Ki67⁺ and BrdU⁺ cells, and this proliferative effect was abrogated by the GZMK mutant or follistatin treatment (Figure 6G). To confirm that the profibrotic effects of GZMK-overexpressing CD8⁺ T cells are directly mediated by these secreted cytokines, we added neutralizing antibodies to the coculture system. As expected, blockade of either Activin A or TGF-β1 significantly reversed the GZMK-OE CD8⁺ T cell-induced upregulation of α-SMA and COL1A1 in fibroblasts (Figure S6D).

Flow cytometry analysis revealed a significant expansion of the GZMK⁺ CD8⁺ T cell population in IPF patients compared to healthy controls (Figure S6E). To determine whether patient-derived CD8⁺ T cells harbor a similar profibrotic capacity, we cocultured HFL1 with CD8⁺ T cells derived from IPF patients. Similar to the GZMK-OE model, the significant upregulation of *α-SMA* and *COL1A1* induced by IPF CD8⁺ T cells was substantially attenuated by neutralizing antibodies against either Activin A or TGF-β1 (Figure 6H). Because Activin A and TGF-β1 signal through the ALK4 and ALK5 receptors respectively, we knocked down these receptors in IPF fibroblasts (Figure 6I). Strikingly, silencing either ALK4 or ALK5 in fibroblasts largely suppressed the upregulation of α-SMA and COL1A1 expression induced by GZMK-OE CD8⁺ T cells (Figure 6J).

Together, these findings provide direct functional evidence that GZMK-overexpressing CD8⁺ T cells can drive fibroblast-to-myofibroblast transition and promote fibroblast proliferation through TGF-β signalling, implicating GZMK as a key effector in profibrotic immune-stromal interactions.

### Targeting GZMK alleviates pulmonary fibrosis and suppresses fibroblast activation

To further validate the pathogenic role of GZMK in fibroblast activation and pulmonary fibrosis, we established a BLM-induced pulmonary fibrosis model in GZMK-deficient mice. Histological analysis showed that BLM-treated wild-type (WT) mice developed severe lung fibrosis, characterized by marked destruction of alveolar architecture, inflammatory cell infiltration, and extensive interstitial thickening, whereas GZMK^-/-^ mice exhibited substantially preserved alveolar structures with reduced inflammatory infiltration. Consistently, Masson's trichrome staining revealed pronounced collagen deposition in WT+BLM lungs, which was markedly attenuated in GZMK^-/-^ mice, indicating reduced extracellular matrix accumulation. Immunohistochemical staining further demonstrated strong GZMK expression in fibrotic regions of WT+BLM lungs, which was largely absent in GZMK-deficient mice (Figure 7A). qPCR analysis showed that BLM significantly induced the expression of fibrotic markers, including *α-SMA* and *COL1A1*, in WT mice, whereas this induction was significantly suppressed in GZMK^-/-^ mice (Figure 7B). In parallel, ELISA analysis revealed that circulating GZMK levels were markedly elevated following BLM treatment in WT mice but were significantly reduced in GZMK-deficient mice (Figure 7C). Consistent with these findings, western blot analysis demonstrated that protein levels of α-SMA, Collagen I, and GZMK were robustly increased in WT+BLM lungs, whereas GZMK deficiency markedly attenuated their expression (Figure 7D). Together, these data demonstrate that GZMK is required for the development of BLM-induced pulmonary fibrosis and plays a critical role in fibroblast activation.

To further explore the therapeutic potential of targeting GZMK, we evaluated the effect of bosutinib, a Src/Abl tyrosine kinase inhibitor known to suppress GZMK expression 33. As shown in Figure S6F, bosutinib markedly alleviated BLM-induced lung fibrosis, as evidenced by improved alveolar architecture and reduced collagen deposition, accompanied by decreased GZMK expression in lung tissues. Consistently, bosutinib significantly reduced *GZMK, α-SMA,* and *COL1A1* expression at both the mRNA and protein levels, as well as circulating GZMK levels in a dose-dependent manner (Figure S6G-I). Furthermore, in a PBMC-fibroblast co-culture system, PBMCs derived from IPF patients secreted higher levels of GZMK compared with healthy controls, which was effectively suppressed by bosutinib treatment (Figure S6J-K). Functionally, IPF-PBMCs promoted fibroblast activation, as indicated by increased α-SMA and COL1A1 expression in HFL1 cells, whereas bosutinib markedly attenuated these effects (Figure S6L). Collectively, these findings indicate that bosutinib administration attenuates fibroblast activation and pulmonary fibrosis in these models. These results highlight GZMK's involvement in fibrotic signalling and raise the possibility that targeting this pathway could complement existing antifibrotic strategies.

### Activin A-ALK4 signalling in CD8⁺ T cells drives fibroblast activation via pSmad2-dependent signalling

To determine whether Activin A signalling regulates GZMK expression in CD8⁺ T cells, we isolated peripheral CD8⁺ T cells from IPF patients and inhibited Activin A activity using a neutralizing antibody. qPCR and western blot analysis revealed that blockade of Activin A resulted in a dose-dependent reduction in GZMK at mRNA and protein levels, indicating that Activin A signalling contributes to sustaining GZMK expression in IPF-derived CD8⁺ T cells (Figure 8A-B). Next, we disrupted ALK4, a type I receptor of the TGF-β superfamily, in IPF-derived CD8⁺ T cells. Knockdown of ALK4 significantly reduced GZMK mRNA expression (Figure 8C), suggesting that Activin A regulates GZMK expression through ALK4-dependent signalling.

To explore downstream signalling in fibroblasts, we established an IPF-derived CD8⁺ T cell-HFL1 co-culture system. Notably, knockdown of ALK4 or GZMK in CD8⁺ T cells, as well as pharmacological inhibition of TGFβR1/ALK4 with TEW, significantly attenuated the expression of these fibrotic markers in fibroblasts (Figure 8D). Western blot showed that co-culture increased Smad2 phosphorylation (pSmad2) expression in fibroblasts, whereas ALK4 and GZMK knockdown, and TEW treatment reduced pSmad2, accompanied by decreased Collagen I and α-SMA protein levels (Figure 8E). Collectively, these findings support a model in which Activin A-ALK4 signalling sustains GZMK expression in CD8⁺ T cells, while GZMK⁺CD8⁺T cells promote fibroblast activation through Smad2-dependent signalling, thereby supporting the existence of a feed-forward immune-stromal circuit that drives fibrosis.

### Preventive TEW and FTY720 treatment protects against bleomycin-induced lung fibrosis

In a bleomycin (BLM)-induced mouse model of pulmonary fibrosis, we tested preventive interventions with TEW and FTY720 using the dosing schedule shown in Figure 8F. To evaluate whether early lymphocyte trafficking contributes to the pulmonary accumulation of GZMK⁺CD8⁺ T cells and subsequent fibrotic remodelling, we included FTY720, a sphingosine 1-phosphate (S1P) antagonist, leading to trap CD8^+^ T cells in lymph nodes and blocking their circulation to the lung 34. Prophylactic administration of TEW or FTY720 improved survival compared with BLM alone (Figure 8G).

qPCR and Western blot analyses of lung tissues consistently showed that TEW markedly reduced the expression of fibrotic markers, including α-SMA and COL1A1, whereas FTY720 primarily decreased GZMK and partially reduced α-SMA, with minimal effect on COL1A1 levels (Figure 8H and 8J). BLM exposure increased circulating GZMK levels, whereas preventive treatment with either FTY720 or TEW reduced GZMK as quantified by ELISA (Figure 8I). Histological assessment via H&E and Masson's trichrome staining revealed marked attenuation of alveolar disruption and collagen deposition in mice with TEW and FTY720 treatment. Additionally, immunohistochemical staining revealed reduced ALK4 and GZMK expression following TEW and FTY720 administration (Figure 8K).

Together, these data indicate that early blockade of lymphocyte egress (FTY720) reduces the pulmonary GZMK signal and alleviates fibrosis, supporting a contribution of infiltrating lymphocytes to GZMK accumulation, while prophylactic treatment with TEW attenuates BLM-induced fibrotic remodelling.

## Discussion

IPF is increasingly recognized as a disease shaped not only by aberrant epithelial repair and fibroblast activation, but also by persistent immune dysregulation across tissue compartments. Here, by integrating paired single-cell transcriptomic and TCR profiling of lung, mediastinal lymph node and peripheral blood samples from a specific group of patients with end-stage IPF, together with in vitro functional assays and in vivo validation, we characterize a GZMK⁺CD8⁺ T cell subset that is enriched in fibrotic lung tissue, clonally linked to lymph node-derived CD8_HSPA1A cells, and functionally capable of promoting fibroblast activation through TGF-β1/Activin A signalling (Figure 9). These findings extend current understanding of immune involvement in IPF by implicating a distinct inflammatory CD8⁺ T-cell programme in the regulation of fibroblast behaviour.

Previous studies have reported increased CD8⁺ T-cell infiltration in IPF lungs and have suggested a role for adaptive immunity in disease progression 35,36. However, the functional heterogeneity within the CD8⁺ compartment has remained poorly characterized. Our single-cell analysis reveals that the CD8⁺ T-cell landscape in IPF is not dominated by highly cytotoxic populations, but instead by a GZMK-expressing subset with reduced cytotoxicity and increased inflammatory and exhaustion-associated features. This observation is consistent with recent reports in cancer and chronic inflammatory conditions, where GZMK⁺CD8⁺ T cells have been described as a distinct population associated with tissue inflammation rather than direct cytotoxicity 14,37,38. Our data extend these observations to IPF and further link this cell state to fibrotic stromal activation.

Our cross-tissue analysis also provides a potential explanation for the long-recognized but poorly understood phenomenon of MLNE in IPF. By combining TCR repertoire analysis and trajectory inference, we found that lung CD8_GZMK cells are clonally and transcriptionally connected to a stress-responsive CD8_HSPA1A population enriched in mediastinal lymph nodes. In this context, the high expression of HSPA1A/HSP70-related genes is notable, because heat shock proteins have been implicated in T-cell trafficking, activation and inflammatory adaptation 39,40. These observations support that lymph nodes may serve as sites of T-cell priming or reprogramming that seed the lung with profibrotic effector populations in IPF, which provides a potential cellular explanation for the clinical association between MLNE and worse disease severity.

Recent work has established that fibroblast heterogeneity is a defining feature of IPF and other fibrotic tissues, with CTHRC1⁺ fibroblasts representing a highly activated matrix-producing population 31,32. Consistent with these reports, we found that FIB_CTHRC1 cells exhibited the strongest fibrotic programme and occupied the terminal end of the fibroblast pseudotime trajectory. Cell-cell communication analysis suggested that CD8_GZMK cells may interact with these fibroblasts through TGF-β family signalling pathways, while fibroblasts may in turn recruit CD8_GZMK cells via chemokine axes such as CXCL14-CXCR4 and CXCL16-CXCR6. These observations are in line with emerging evidence that fibroblasts actively participate in immune cell recruitment and niche formation in IPF, rather than serving solely as downstream effector cells.

Our functional data suggest that the profibrotic activity of GZMK⁺CD8⁺ T cells is mediated less by direct cytotoxicity than by paracrine inflammatory signalling. This interpretation is consistent with the broader re-evaluation of granzyme biology, in which granzymes are increasingly recognized as mediators of extracellular and noncytotoxic functions rather than exclusive executors of target-cell killing 41. For example, extracellular GZMK has been shown to activate endothelial cells and induce inflammatory cytokine production through protease-activated receptor-1, supporting a role for GZMK in shaping tissue inflammation beyond canonical cytolysis 42. Our results including direct GZMK stimulation, which exhibited dose-responsive effects, along with ligand neutralization and receptor-specific knockdown that reduced the expression of profibrotic genes, suggested that GZMK may serve as a potential driver of downstream profibrotic effects.

A key mechanistic insight from our study is the implication of Activin A-ALK4 signalling as a central pathway linking GZMK^+^CD8⁺ T cells to fibroblast activation. Activin A is a TGF-β superfamily cytokine with broad roles in immunity, tissue repair and fibrosis 43. Prior studies have shown that activated CD4⁺ T cells secrete Activin A in an autocrine manner through the ALK4 receptor to activate ERK signalling, thereby promoting their differentiation into pathogenic Th17 cells and enhancing their inflammatory function in autoimmune neuroinflammation 44. In the tumour microenvironment, Activin A has been shown to induce its own expression (INHBA) and sustain inflammatory activation in macrophages through ALK-dependent signalling, thereby reinforcing cytokine production and establishing a protumoral feed-forward loop 45. Here, we found that GZMK-modulated CD8⁺ T cells altered INHBA and TGFB1 expression, and that blockade of Activin A or disruption of ALK4 reduced GZMK expression in CD8⁺ T cells. In parallel, fibroblasts co-cultured with CD8⁺ T cells showed increased Smad2 phosphorylation and fibrotic marker expression, both of which were attenuated by ALK4 or GZMK knockdown in immune cells or by TEW treatment. Together, these findings support a feed-forward model in which Activin A-ALK4 signalling sustains a profibrotic GZMK⁺CD8⁺ T cell state, while immune-derived TGF-β family signals activate fibroblast Smad2 signalling and matrix production.

This mechanistic framework also has therapeutic implications. Current first-line IPF therapies, pirfenidone and nintedanib, can slow disease progression but do not reverse established fibrosis and do not specifically target the immune programmes that may sustain fibroblast activation 46,47. TGF-β-directed strategies, including integrin-based approaches, are under active investigation 48,49, but receptor-level blockade has not yet been established in IPF. TEW-7197 (vactosertib) is a potent inhibitor of ALK4 and TGFβR1 that suppresses Smad2/3 activation and has shown antifibrotic or anti-remodelling potential in other contexts 50-52. In our study, TEW attenuated fibroblast activation in vitro and reduced fibrotic remodelling in vivo, supporting the translational relevance of this pathway. At the same time, bosutinib-mediated suppression of GZMK-associated responses and genetic GZMK deficiency both alleviated fibrosis in the bleomycin model, further supporting this immune-stromal circuit as therapeutically actionable. These results raise the possibility that targeting either the pathogenic CD8⁺ T cell state or the downstream ALK4/TGFβR1 signalling axis could complement existing antifibrotic strategies.

Several limitations should be acknowledged. First, our single-cell cohort included only four male IPF patients undergoing lung transplantation, all of whom had a history of smoking and presented with mediastinal lymph node enlargement. Therefore, it does not capture the full clinical and molecular heterogeneity of IPF or allow robust stratification by sex, smoking burden, or disease stage. Future investigations will not only require larger and more diverse cohorts, including female patients and clinically defined subgroups, to confirm the generalizability of our findings, but also could include lineage-tracing or adoptive transfer experiments to provide definitive evidence of developmental directionality. Second, although our data support a role for Activin A-ALK4 signalling in maintaining GZMK expression, the precise molecular mechanisms linking this pathway to GZMK transcription remain to be defined. Finally, given the potential pleiotropic effects of these pharmacological interventions, future directions should also focus on developing highly selective inhibitors, so as to further assess the clinical feasibility of targeting this signalling axis.

In conclusion, our study highlights a GZMK⁺CD8⁺ T cell population as a relevant immune component contributing to fibroblast activation in advanced IPF and implicates TGFβ1/Activin A signalling as a central pathway linking immune and stromal compartments. These findings provide new insight into the immunopathogenesis of IPF and highlight potential targets for therapeutic intervention aimed at disrupting pathogenic immune-stromal interactions.

## Supplementary Material

Supplementary figures and table.

## Figures and Tables

**Figure 1:**
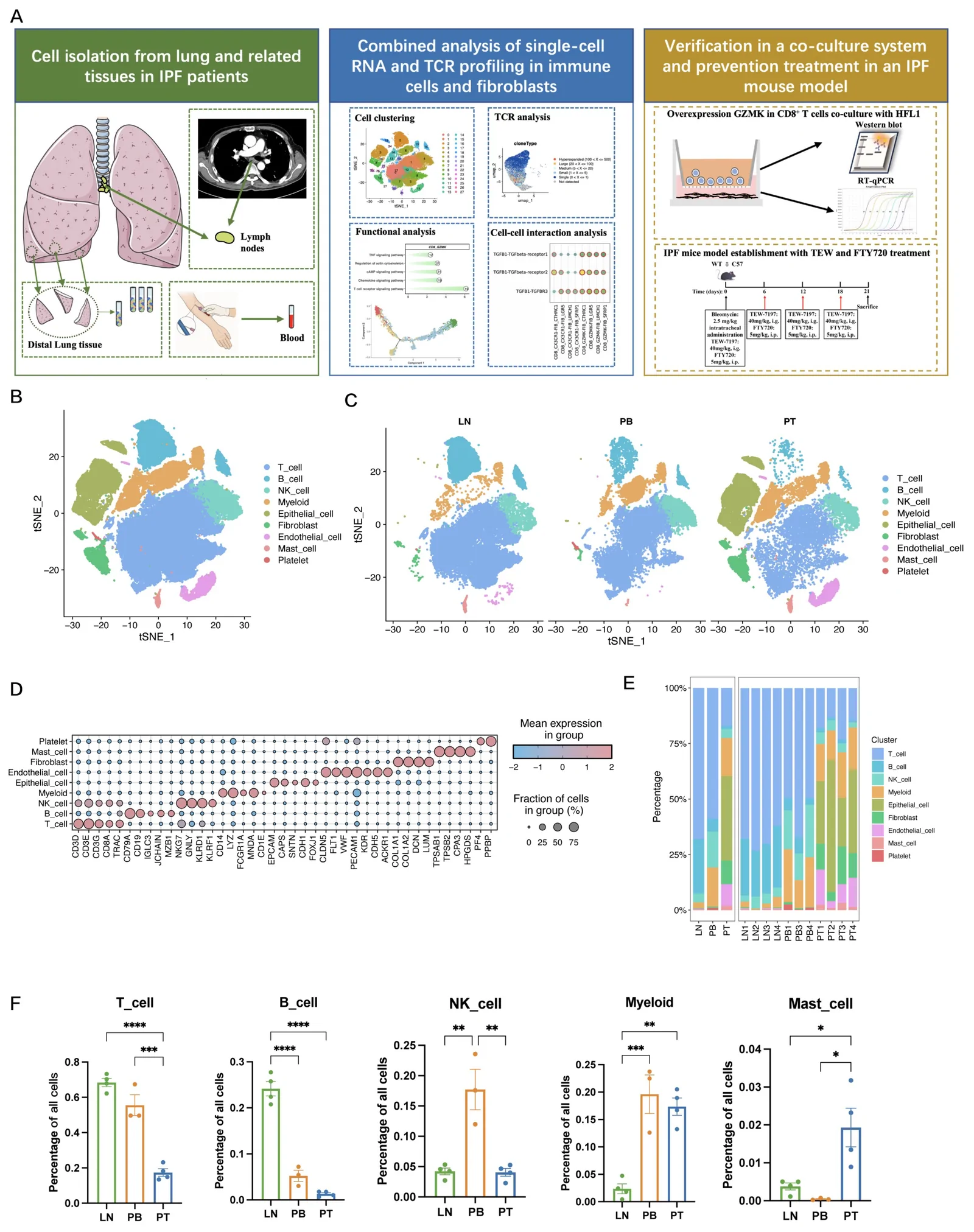
** Single-cell landscape of immune and stromal cell composition in IPF lungs, lymph nodes, and peripheral blood. A.** Overall study design diagram with 11 IPF samples constructed by using the 10X Genomics Platform. **B.** t-SNE plot showing 9 major cell types of 83,319 cells. Each dot represents an individual cell, coloured according to its assigned cell type on the basis of transcriptional signatures. **C.** t-SNE plots showing the distribution of major cell populations across different sample sites, including lymph nodes (LN), peripheral blood (PB), and pulmonary tissues (PT) from IPF patients. **D.** Dot plot showing marker gene expression for each cell type, with colour intensity indicating average expression levels and dot size representing the proportion of cells expressing each marker gene, as shown in the scale bar on the right. **E.** The left panel shows the distribution of cell types across different tissue categories, including LN, PB, and PT samples. The right panel illustrates the cell type composition within individual samples from these tissue categories. **F.** Bar plot showing the percentages of T cells, B cells, NK cells, myeloid cells, and mast cells across the LN, PB, and PT compartments in IPF patients (LN, n = 4; PB, n = 3; PT, n = 4). The data are presented as the mean ± standard error of the mean. Statistical significance was determined using a one-way ANOVA with multiple comparisons. *P<0.05, **P<0.01, ***P<0.001, ****P<0.0001.

**Figure 2:**
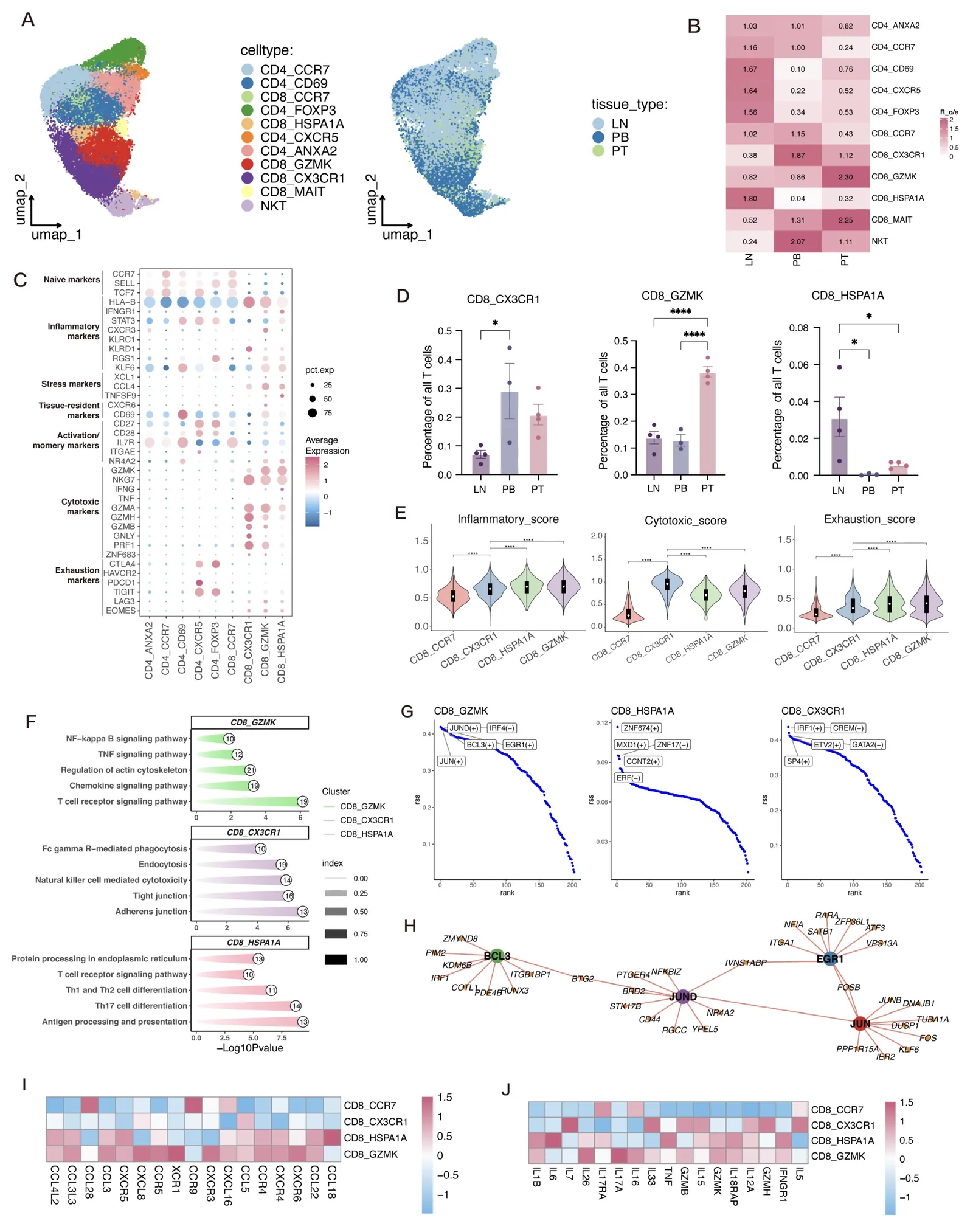
** Proinflammatory GZMK^+^CD8^+^ T cells are enriched in IPF lung tissues. A.** UMAP plot of T cells from IPF patients, with clustering into 11 distinct clusters, each represented by a different colour (left) and their distribution across different sample sites, including LN, PB, and PT (right). **B.** Heatmap displaying the tissue preference of each T cell subset across different tissue compartments in IPF patients analysed by Ro/e. **C.** Dot plot showing the expression of functional marker genes across CD8^+^ T cell subsets. **D.** Bar plot showing the percentages of CD8_GZMK, CD8_HSPA1A, and CD8_CX3CR1 cells across the LN, PB, and PT compartments in IPF patients (LN, n = 4; PB, n = 3; PT, n = 4). Data are presented as mean ± standard error of the mean. Statistical significance was determined using a one-way ANOVA with multiple comparisons. *P<0.05, ***P<0.001, ****P<0.0001. **E.** Violin plot showing the inflammatory score, cytotoxic score, and exhaustion score across CD8⁺ T cell subsets. **F.** KEGG pathway enrichment results for the CD8_GZMK, CD8_HSPA1A, and CD8_CX3CR1 subsets based on the top genes from each cluster.** G.** SCENIC regulatory-signature ranking curves for CD8_GZMK, CD8_HSPA1A, and CD8_CX3CR1. **H.** SCENIC-derived transcription factor network linking the four most influential regulons in the CD8_GZMK subset (BCL3, JUND, JUN, and EGR1) to their top target genes. **I.** Heatmap showing the expression of chemokine and chemokine-receptor genes across CD8^+^ T cell subsets. **J.** Heatmap displaying the expression of proinflammatory cytokine genes in CD8^+^ T cell subsets.

**Figure 3:**
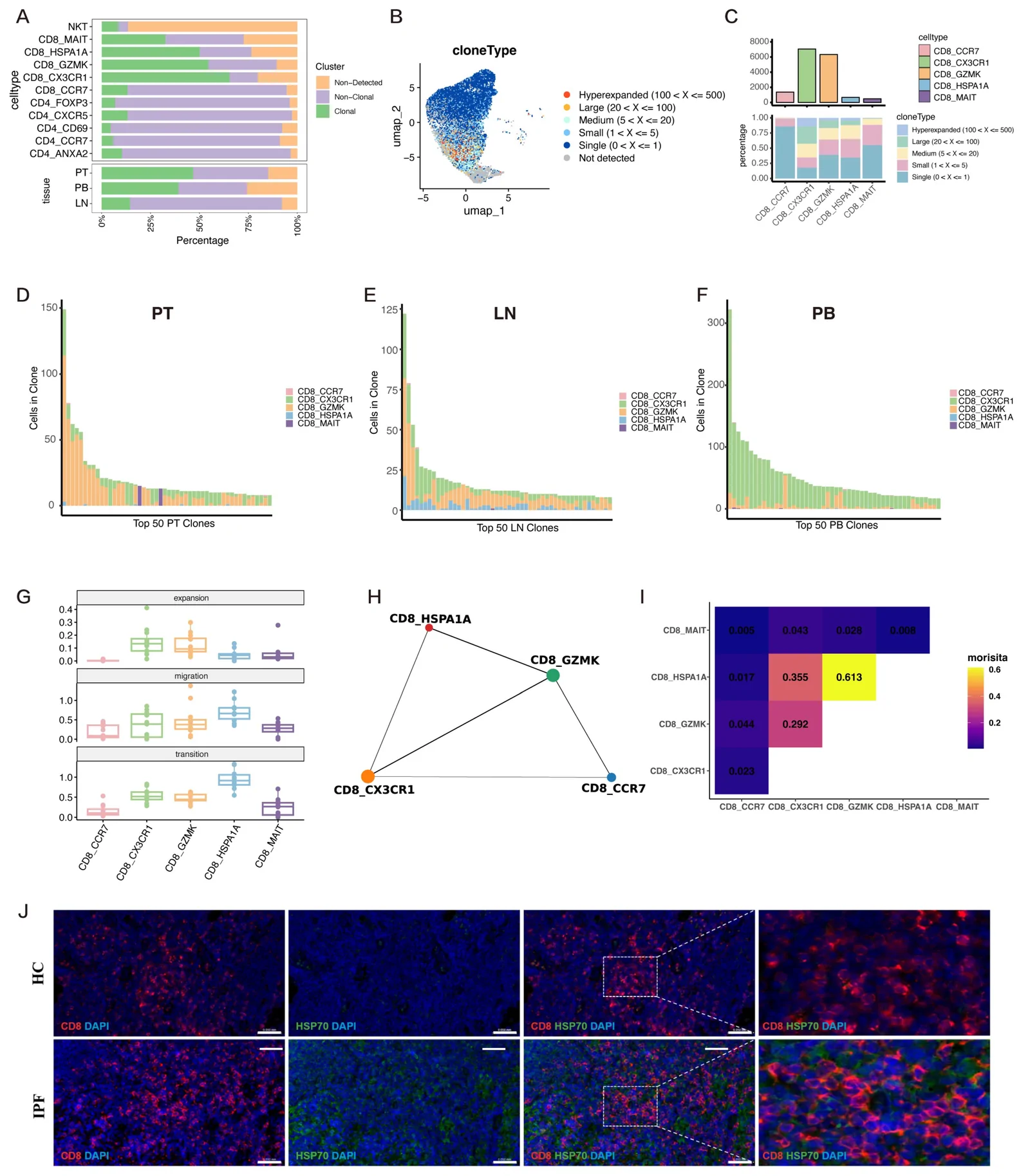
** TCR analysis revealed a close relationship between GZMK^+^CD8^+^ T cells and HSPA1A^+^CD8^+^ T cells. A.** Percentages of clonal, non-clonal, and non-detected T cells across different subsets and tissues in IPF. **B.** UMAP visualization displaying the distribution of T cell clonotypes on the basis of their clone sizes. **C.** Cell numbers and clonotypes of major CD8⁺ T cell subsets in IPF. **D‒F.** Bar plots showing the sizes and cluster composition of the top 50 clones, along with the clone size distributions for the pulmonary tissues (D), lymph nodes (E), and peripheral blood (F). **G.** Clonal expansion, migration, and transition potential of CD8^+^ T cells assessed using STARTRAC indices. **H.** PAGA analysis illustrating CD8^+^ T cell clusters, where each dot corresponds to a specific T cell cluster. **I.** Heatmap showing the TCR clonal similarity between CD8^+^ T cell subsets derived from the TCR analysis. **J.** Representative multiplex immunofluorescence images of lymph node sections from healthy controls (HC) and idiopathic pulmonary fibrosis (IPF) patients stained for CD8 (red), HSP70 (green), and DAPI (blue). Scale bar, 50 μm.

**Figure 4:**
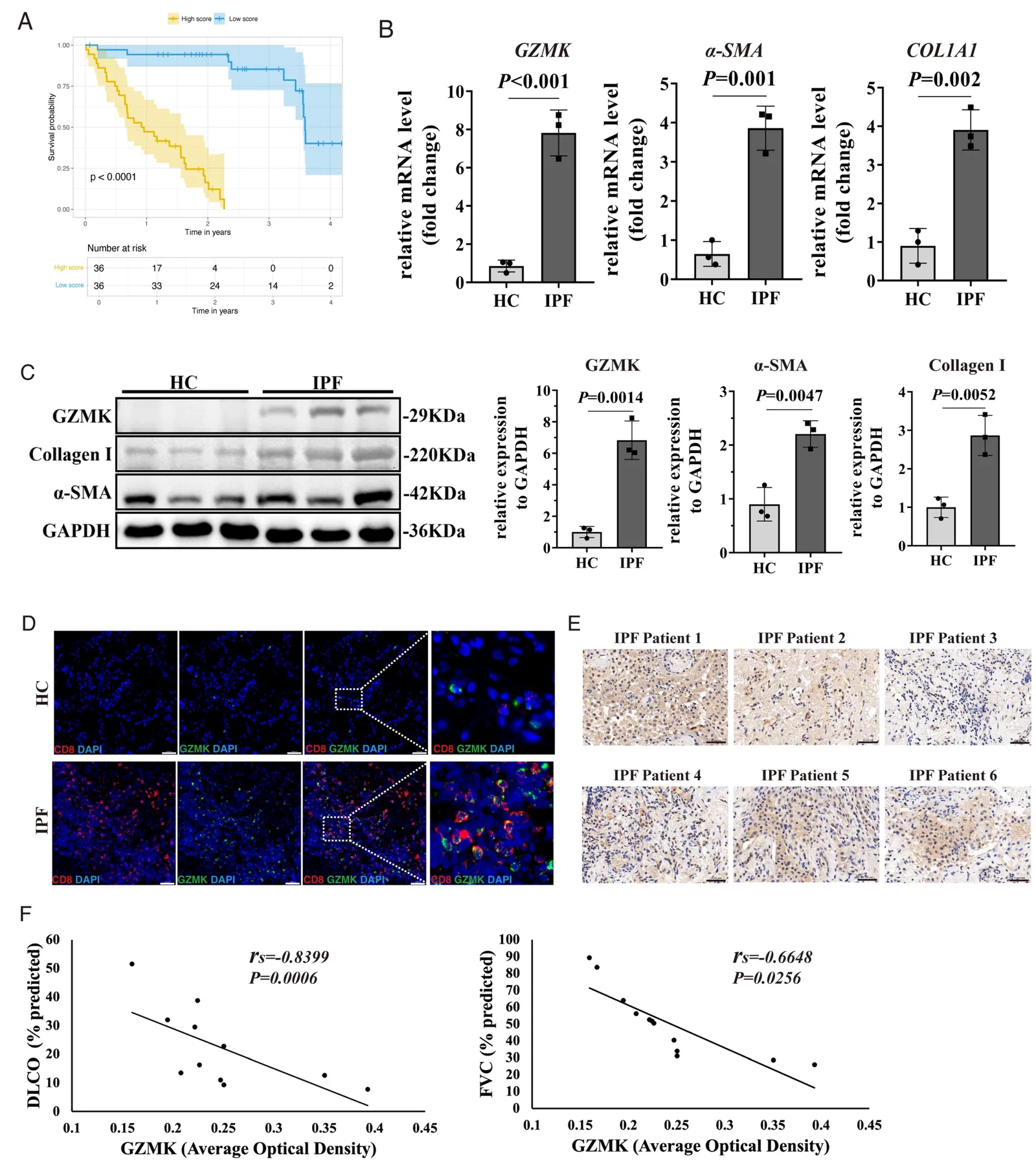
** GZMK⁺ CD8⁺ T cells are elevated in IPF and are associated with fibrosis severity and poor prognosis. A.** Kaplan-Meier survival curve analysis of the GSE28042 dataset revealed that IPF patients with higher GZMK⁺CD8⁺ T cell scores had significantly worse overall survival (OS). **B.** qPCR analysis showing significantly increased mRNA expression of *COL1A1*, *α-SMA*, and *GZMK* in lung tissues from IPF patients compared with those from healthy controls (n=3). **C.** Western blot analysis revealed markedly elevated protein levels of Collagen I, α-SMA, and GZMK in lung tissues of patients with IPF compared with those in HC, with GAPDH used as a loading control (n=3). Data are presented as mean ± standard error of the mean (SEM). Statistical significance was determined using two-tailed Student's t-tests. **D.** Multiplex immunofluorescence staining showing enrichment of CD8⁺GZMK⁺ double-positive cells (red and green) in IPF lung tissue, but rarely in normal lung tissue. DAPI (blue) was used to stain the nuclei. Scale bar, 50 μm. **E.** Immunohistochemical staining of GZMK in lung tissue sections from six independent IPF patients. Brown signals indicate positive staining for GZMK and nuclei were counterstained with haematoxylin (blue). Scale bar, 50 μm. **F.** Scatter plots showing the relationship between GZMK expression (quantified as average optical density) and lung function parameters, including DLCO (% predicted) (left) and FVC (% predicted) (right) (n=12). Spearman's correlation coefficients (r_s_) and corresponding p values are indicated in each panel.

**Figure 5:**
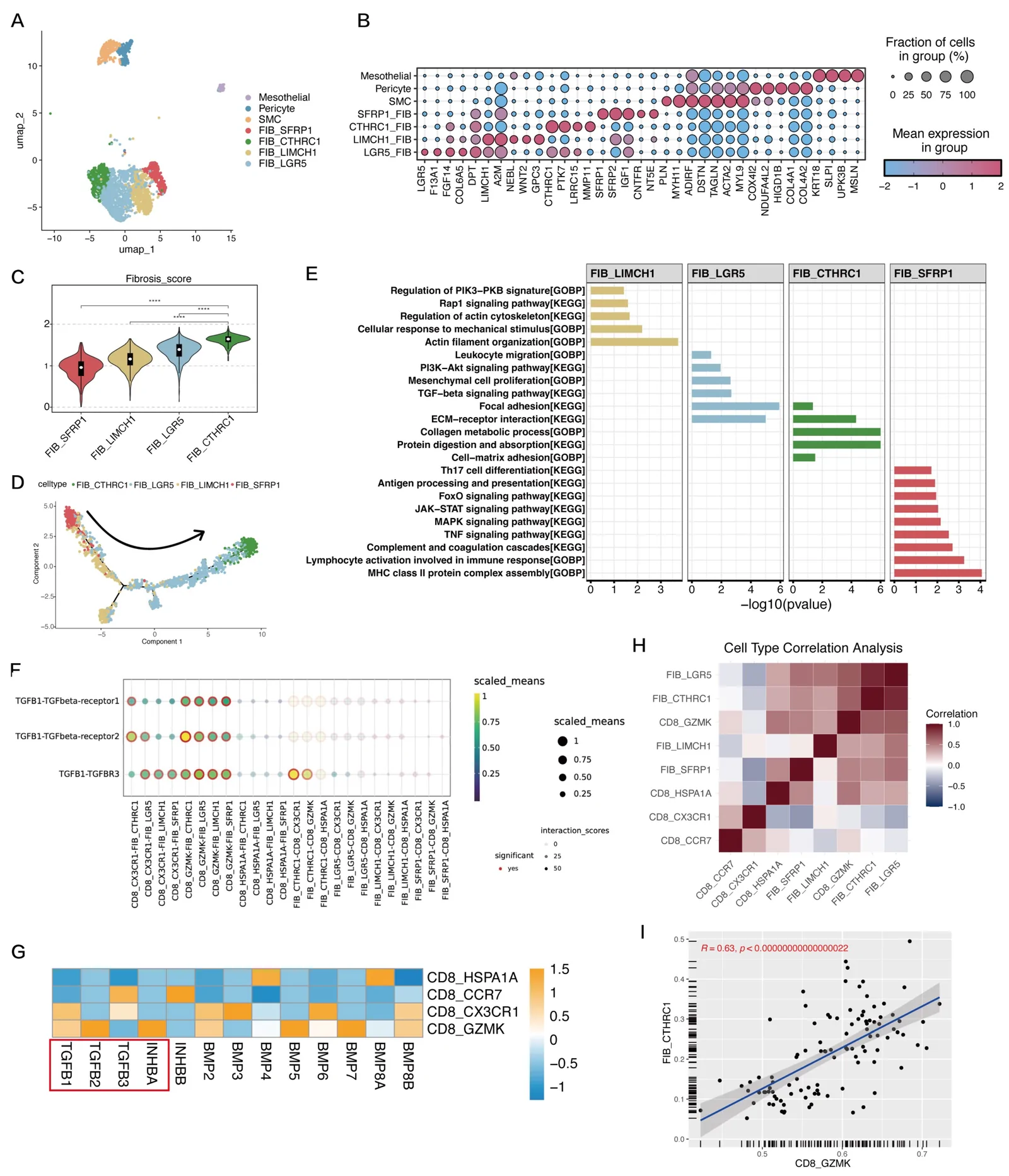
** GZMK^+^CD8^+^ T cell‒fibroblast crosstalk drives fibrogenesis signalling in IPF. A.** UMAP plot of fibroblasts from IPF patients, with reclustering into 7 distinct clusters, each represented by a different colour. **B.** Dot plot displaying the expression of marker genes across identified fibroblast clusters. **C.** Violin plot showing fibrosis scores across fibroblast subsets. **D.** Monocle analysis revealing the differentiation trajectories of fibroblast subtypes in IPF. **E.** Bar plot displaying the selected KEGG and GO pathway enrichment results for four fibroblast subsets based on the top 200 genes from each cluster. **F.** Ligand-receptor interaction analysis revealed significant TGFB1-TGFBR1/2/3 signalling interactions between CD8_GZMK subsets and fibroblast populations in IPF lung tissue. **G.** Heatmap showing the expression profiles of TGF-β superfamily ligands (including TGFBs, INHBs, and BMPs) across CD8⁺ T cell subsets. **H.** Heatmap of cell-type correlations based on ssGSEA analysis showing a strong positive correlation between CD8_GZMK cells and the FIB_CTHRC1 fibroblast subset. **I.** Correlation scatter plot of the CD8_GZMK and FIB_CTHRC1 subsets revealed a significant positive correlation, with a Spearman correlation coefficient of 0.63.

**Figure 6:**
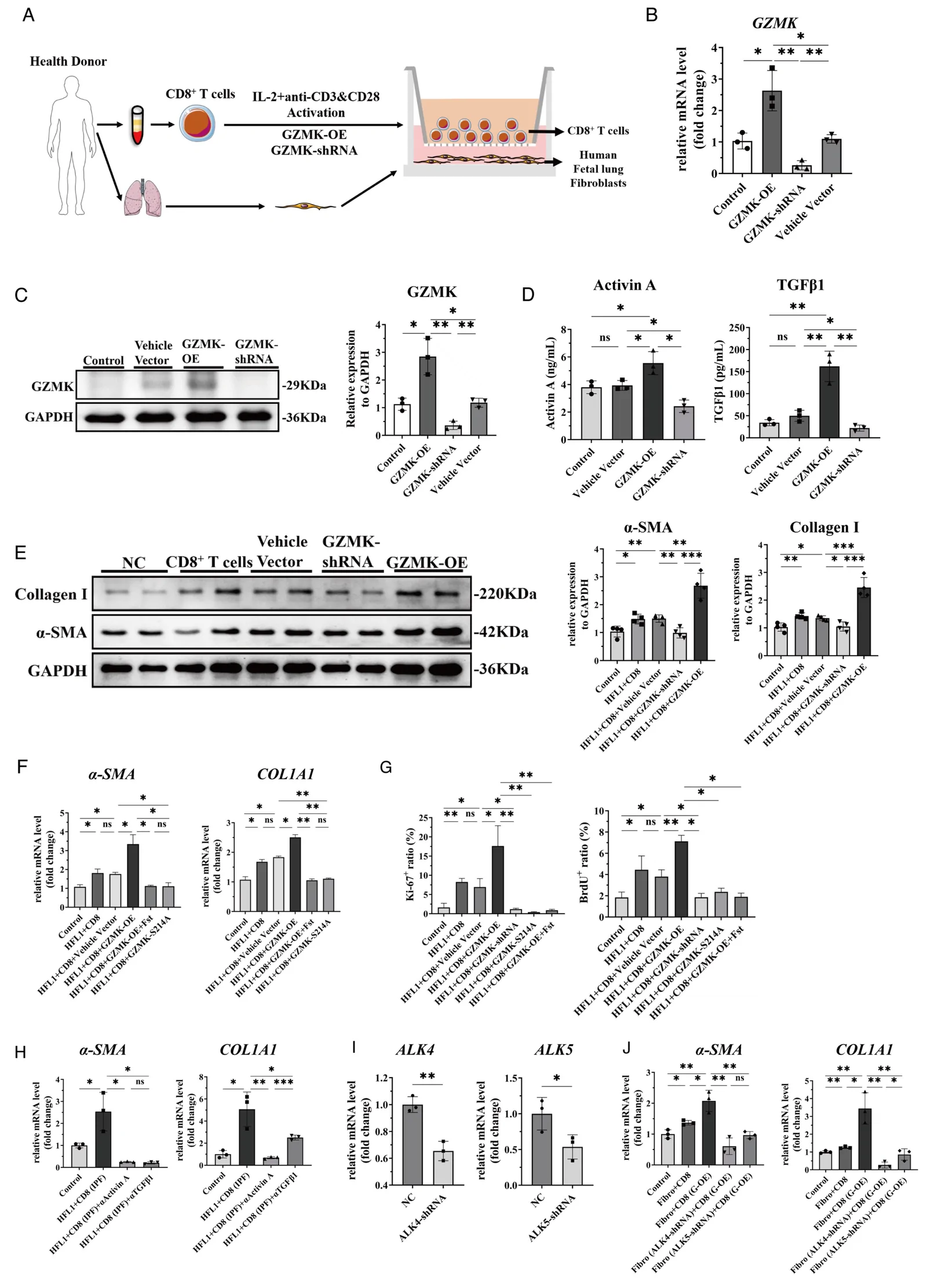
** GZMK overexpressed CD8⁺ T cells promote fibroblast-to-myofibroblast transition and proliferation in a coculture system. A.** Experimental workflow: Human foetal lung fibroblasts (HFL1) were cocultured with human peripheral CD8⁺ T cells that had been activated and genetically modified to overexpress (OE) or knockdown (shRNA) GZMK to assess functional effects. **B‒C.** qPCR analysis (B) and western blotting (C) confirming the successful overexpression and knockdown of GZMK in human CD8⁺ T cells after transduction (n=3). **D. ELISA analysis of Activin A and TGFβ1 secretion in GZMK-modified CD8⁺ T cells** (n=3). **E.** Western blot analysis of collagen I and α-SMA expression in fibroblasts cocultured with different types of CD8⁺ T cells (n=3). **F.** qPCR analysis showing the expression levels of *α-SMA*, and *COL1A1* in fibroblasts cocultured with CD8⁺ T cells under the different conditions (n=3). Fst, follistatin. **G.** Flow cytometric quantification of Ki67⁺ (left) and BrdU^+^ (right) fibroblasts after coculture with CD8⁺ T cells under the different conditions (n=3). **H.** qPCR analysis of *α-SMA* and *COL1A1* expression in HFL1 cocultured with IPF-derived CD8⁺ T cells in the presence of neutralizing antibodies against Activin A (αActivin A) or TGF-β1 (αTGFβ1) (n=3). **I.** qPCR validation of ALK4 and ALK5 knockdown (shRNA) in IPF fibroblasts (n=3). **J.** qPCR analysis of *α-SMA* and *COL1A1* expression in IPF fibroblasts with or without ALK4 or ALK5 knockdown following coculture with control or GZMK-overexpressing (G-OE) CD8⁺ T cells (n=3). Data are presented as mean ± standard error of the mean (SEM). Statistical significance was determined using a one-way ANOVA with multiple comparisons. *P<0.05, **P<0.01, ***P<0.001, ****P<0.0001, ns, not significant.

**Figure 7:**
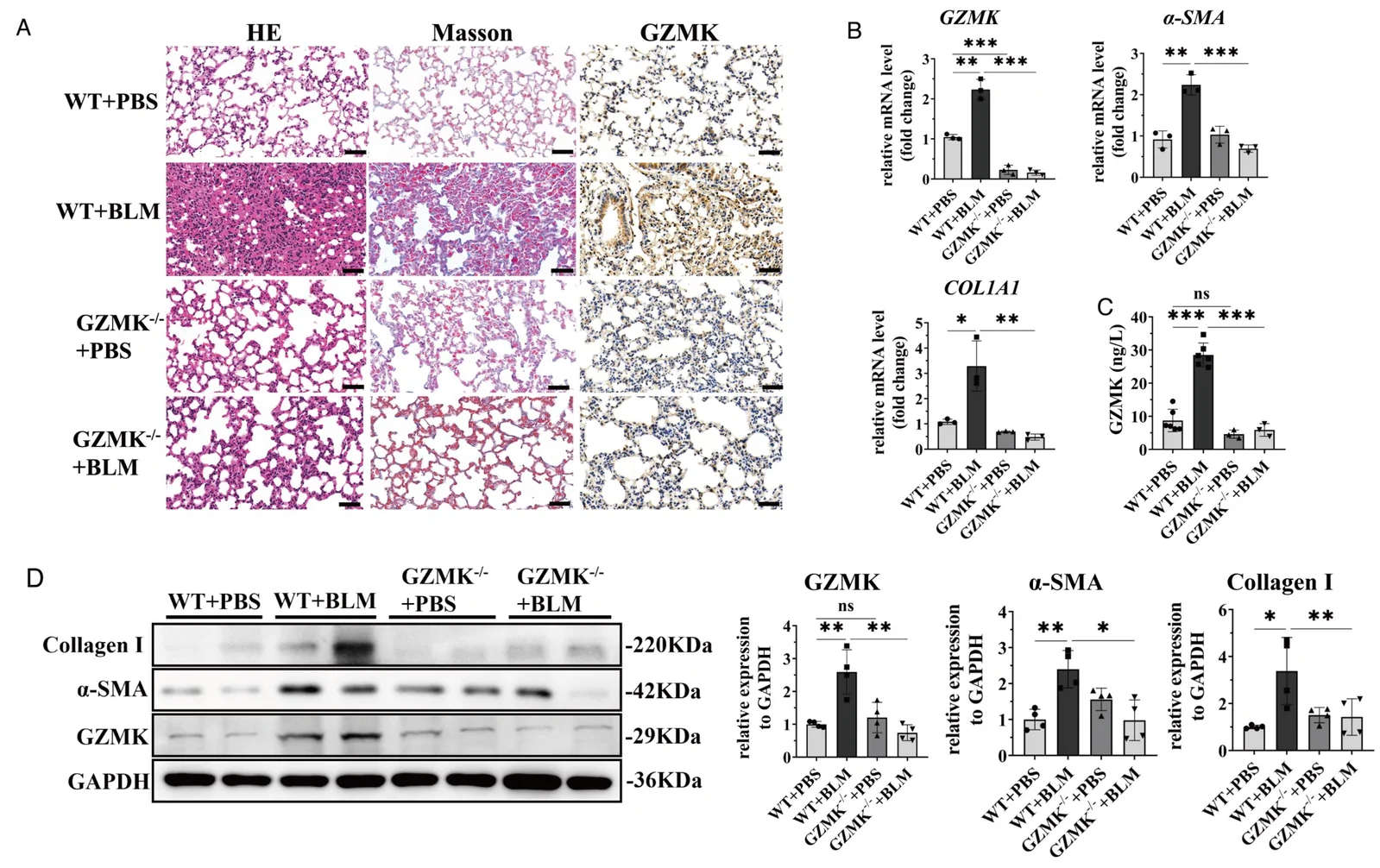
** Knockout of GZMK attenuates pulmonary fibrosis in BLM-induced mice. A.** Bleomycin (BLM)-induced lung fibrosis mice in GZMK homozygous knockout mice. Representative images of HE, Masson's trichrome, and immunohistochemical staining for GZMK in lung tissues from different groups. Scale bar, 50μm. **B.** Genes expression determined by qPCR analysis showed significantly decreased level of *GZMK*, *COL1A1*, and *α-SMA* in BLM-treated GZMK homozygous knockout mice compared to BLM-induced WT mice (n=3). **C.** Serum GZMK protein levels in the indicated GZMK homozygous knockout mice groups were quantified by ELISA (n≥3). **D.** Western blot analysis of GZMK, Collagen I, and α-SMA protein levels in lung tissue homogenates from the indicated groups (n=4). Data are presented as mean ± standard error of the mean (SEM). Statistical significance was determined using a one-way ANOVA with multiple comparisons. *P<0.05, **P<0.01, ***P<0.001, ****P<0.0001, *ns*, not significant.

**Figure 8:**
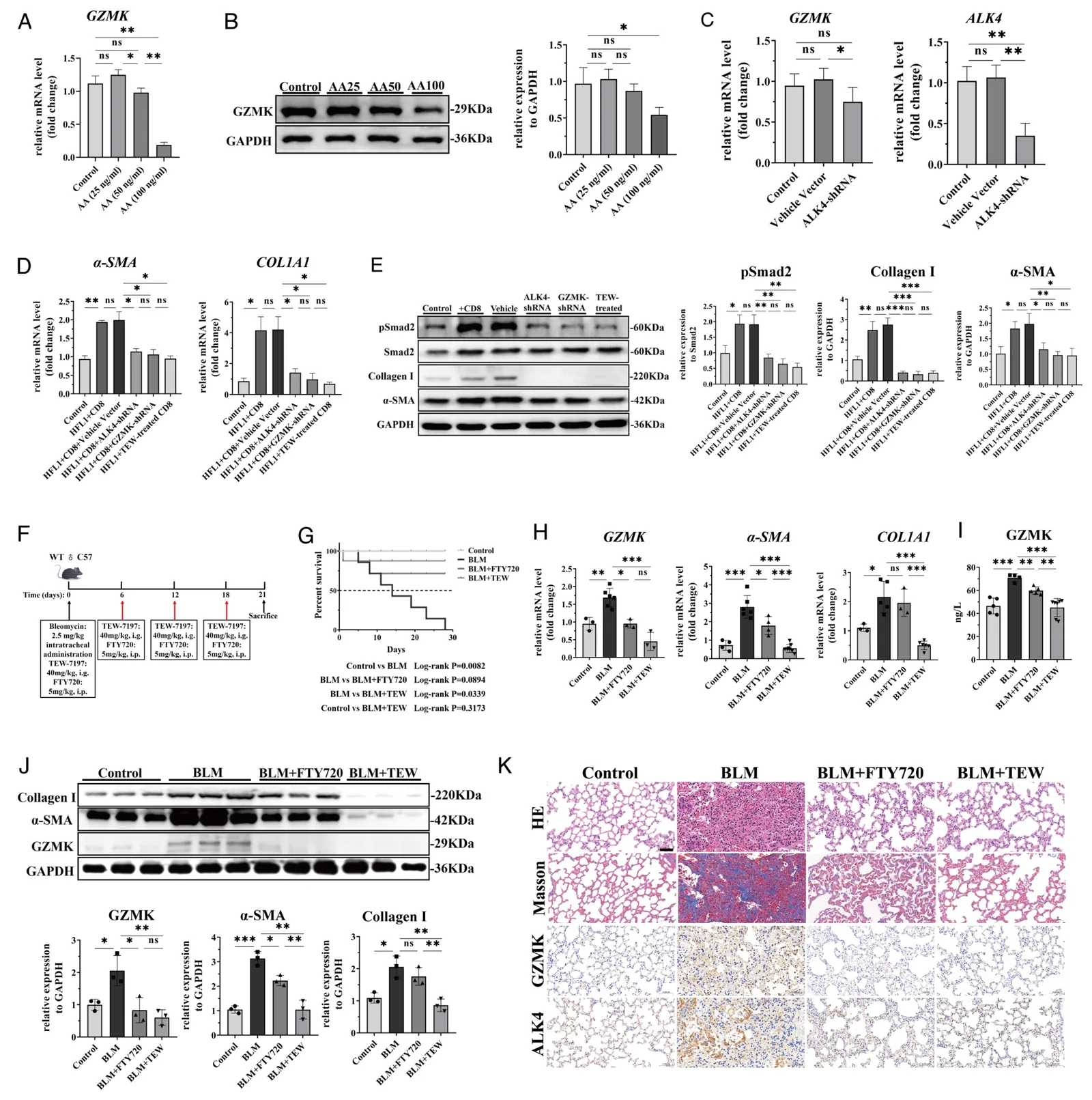
** Activin A-ALK4 signalling in CD8⁺ T cells drives fibroblast activation and pulmonary fibrosis. A.** qPCR analysis showing the expression levels of GZMK in CD8^+^ T cells from PBMCs of IPF patients treated with an anti-Activin A neutralizing antibody (AA) (n=3). **B.** Western blot analysis of GZMK expression in CD8^+^ T cells from PBMCs of IPF patients treated with an anti-Activin A neutralizing antibody (AA) (n=3). **C.** qPCR validation of **ALK4** knockdown in IPF-derived CD8⁺ T cells (right) and **GZMK** mRNA levels were concomitantly reduced in shALK4 CD8⁺ T cells (left) (n=3). **D.** qPCR analysis of ***α-SMA*** and ***COL1A1*** expression in HFL1 fibroblasts following co-culture with IPF-derived CD8⁺ T cells under the indicated conditions (vector control, shALK4, shGZMK, or TEW treatment) (n=3). **E.** Western blot analysis of pSmad2, **Smad2, collagen I,** and **α-SMA in fibroblasts** cocultured with IPF-derived CD8⁺ T cells under the different conditions (n=3). **F.** Schematic of the in vivo experimental timeline. Pulmonary fibrosis was induced in C57BL/6 mice via intratracheal instillation of bleomycin (2.5 mg/kg). Mice were treated with TEW (TEW-7197, 40 mg/kg, i.g.), FTY720 (5 mg/kg, i.p.) or vehicle at the indicated time points. **G.** Kaplan-Meier survival curves for the indicated treatment groups. **H.** qPCR analysis of fibrotic and target genes (*α-SMA*, *COL1A1*, *GZMK*) in lung tissue from bleomycin-induced mice treated with TEW, FTY720 or vehicle control (n=3). **I.** Serum GZMK concentrations in the indicated treatment groups were quantified by ELISA. **J.** Western blot analysis of collagen I, α-SMA and GZMK protein levels in mouse lung tissue from the indicated treatment groups (n=3). **K.** Representative photomicrographs of lung sections stained with Hematoxylin and Eosin (H&E; top), Masson's Trichrome (middle; collagen in blue), and immunohistochemistry (IHC) for GZMK and ALK4 (bottom). Scale bar, 50 μm. Statistical significance was determined using a one-way ANOVA with multiple comparisons. *P<0.05, **P<0.01, ***P<0.001, ****P<0.0001, *ns*, not significant.

**Figure 9:**
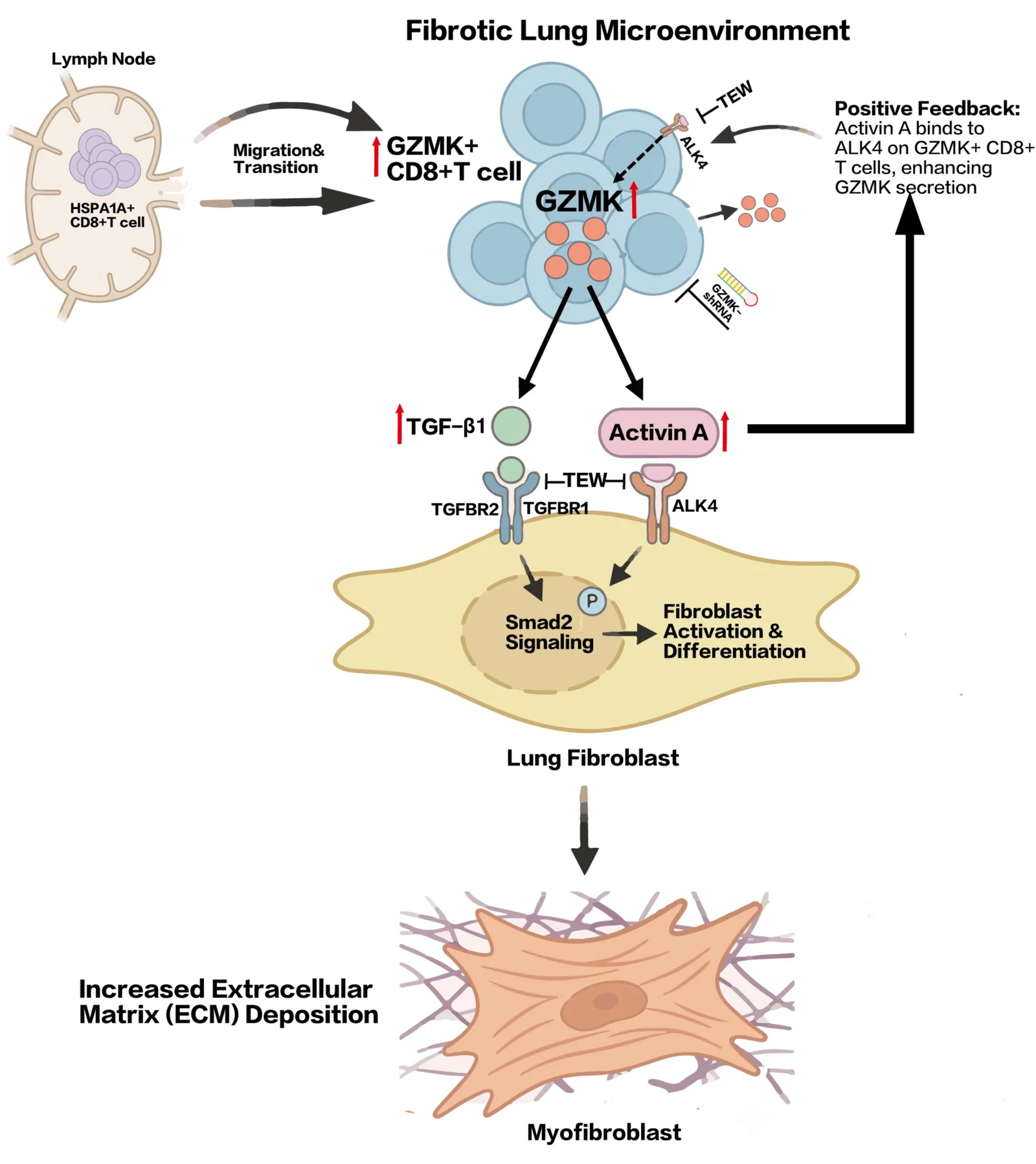
Schematic illustration of how GZMK⁺CD8⁺ T cells promote fibroblast activation in IPF by inducing TGF-β1 and Activin A signalling and amplifying it through an Activin A-mediated autocrine positive feedback loop.

## Data Availability

The scRNA-seq and scTCR-seq data supporting the findings of this study have been deposited at GSA-Human (HRA013103) under accession code PRJCA032809. Previously published microarray data analyzed together are available under accession codes GSE28042, GSE213001, GSE214085 and GSE131907. All other supporting data of this study are available from the corresponding author on reasonable request.

## Abbreviations

IPF: idiopathic pulmonary fibrosis
α-SMA: α-smooth muscle actin
MLNE: mediastinal lymph node enlargement
CT: computed tomography
DLCO: diffusing capacity for carbon monoxide
GZMK: granzyme k
TGF-β: transforming growth factor-β
TCR: t cell receptor
PBMCs: peripheral blood mononuclear cells
UMAP: uniform manifold approximation and projection
UMI: unique molecular identifier
PAGA: partition-based graph abstraction
GO: gene ontology
KEGG: Kyoto encyclopedia of genes and genomes
TFs: transcription factors
HFL1: human fetal lung fibroblasts
qRT-PCR: quantitative real-time PCR
IHC: immunohistochemistry
t-SNE: t-distributed stochastic neighbour embedding
FVC: forced vital capacity
