# Supplementary material for: Integrated single-cell TCR analysis across tissue identifies GZMK+CD8+ T cells promoting fibrosis via TGF-β signalling in IPF
Source: Int J Biol Sci. 2026 Sep 3;22(14):7862–86. doi: 10.7150/ijbs.134974 (PMC13618230; doi:10.7150/ijbs.134974)
Supplement: Supplementary file 1 [file ijbsv22p7862s1.pdf]

Supplementary Figure 1

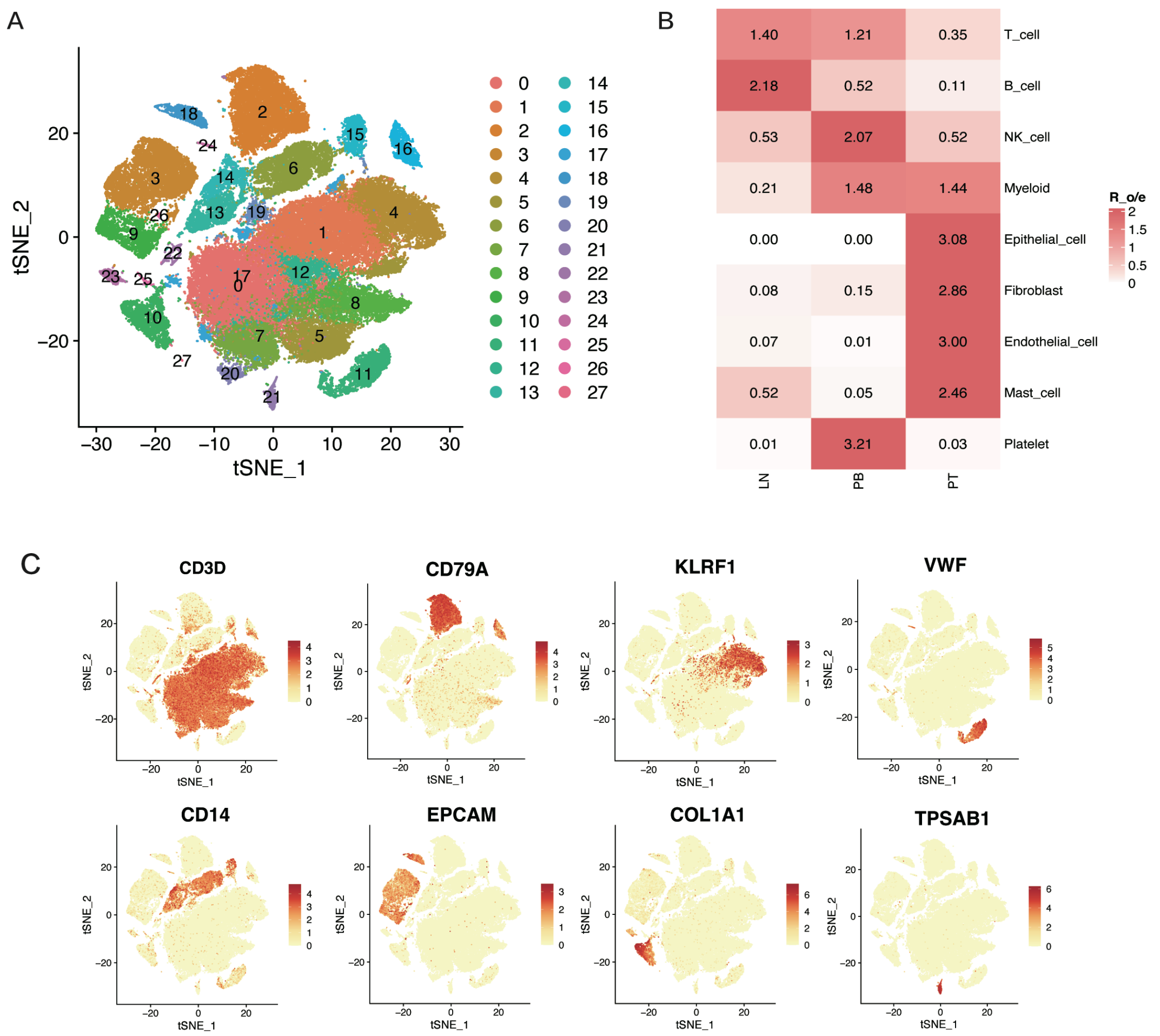

Supplementary Figure 1 Distribution of Major Immune Cell Types Across Different Tissues in IPF Patients.

A. t-SNE plot depicting the transcriptional landscape of single cells, grouped into 28 distinct clusters based on unsupervised clustering analysis. **B.** Tissue preference of each major cluster in IPF estimated by Ro/e. **C.** t-SNE plots showing the expression pattern of marker genes of each identified cell type.

Supplementary Figure 2

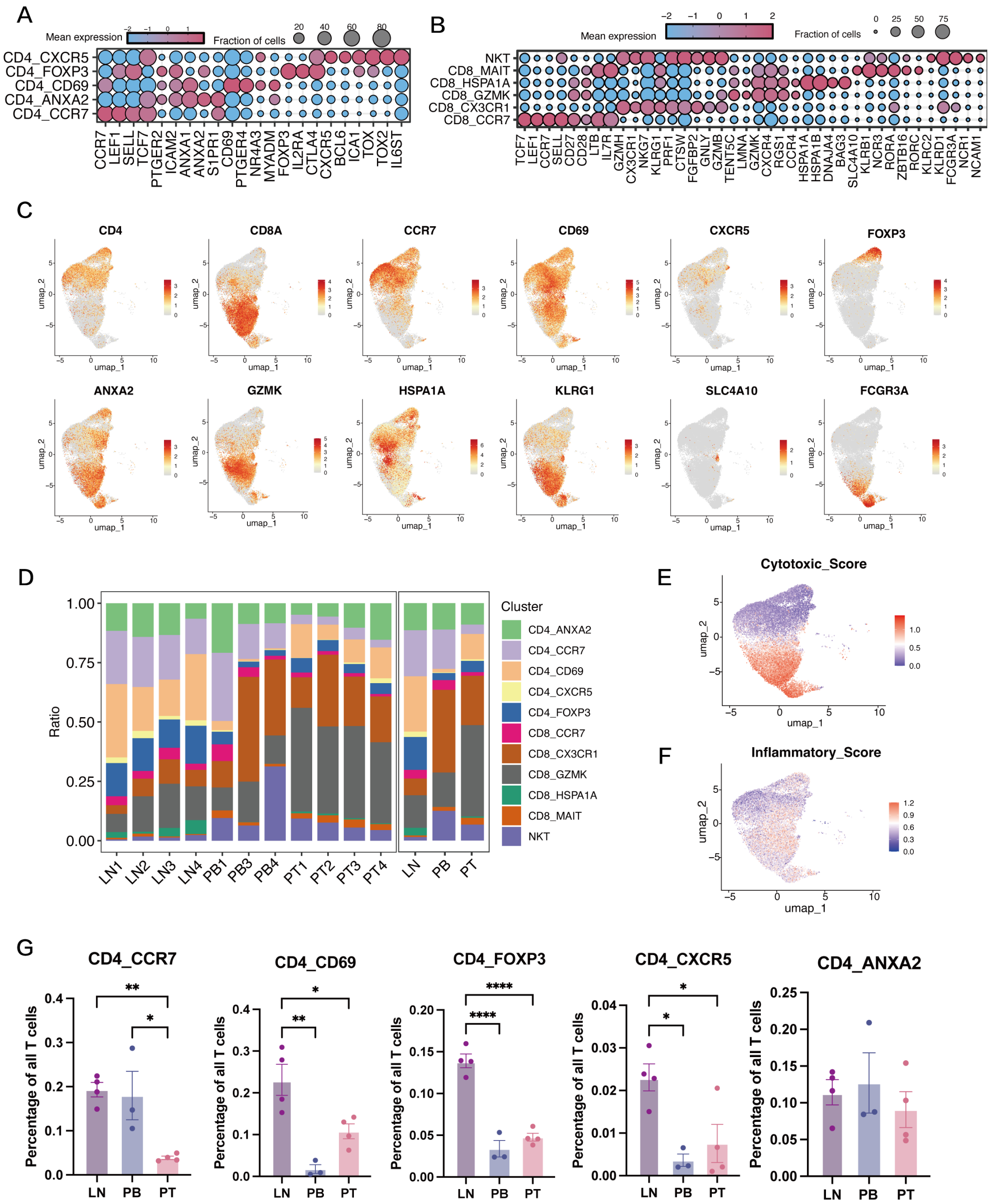

Supplementary Figure 2 Analysis of Marker Expression, Functional Scores, and Subset-Specific DEGs in IPF T Cells.

A. Dot plot displaying the expression of selected marker genes across identified CD4^{+} T cell clusters. **B.** Dot plot displaying the expression of selected marker genes across identified CD8^{+} T cell clusters. **C.** UMAP plots showing the expression pattern of

marker genes of identified cell types. **D.** The right panel shows the proportional distribution of T cell types across different tissue categories, including LN, PB, and PT samples. The left panel illustrates the cell type composition within individual samples from these tissue categories. **E-F.** UMAP visualization showing the distribution of Cytotoxicity_score (E) and Inflammatory_score (F) across different T cell clusters. **G.** Bar plot showing the percentages of CD4_CCR7, CD4_ANXA2, CD4_CD69, and CD4_FOXP3 cells across LN, PB, and PT compartments in IPF patients. Data are presented as mean ± standard error of the mean (SEM). Statistical significance was determined using a one-way ANOVA with multiple comparisons. $^{*}P < 0.05$, $^{**}P < 0.01$, $^{***}P < 0.001$, $^{****}P < 0.0001$.

Supplementary Figure 3

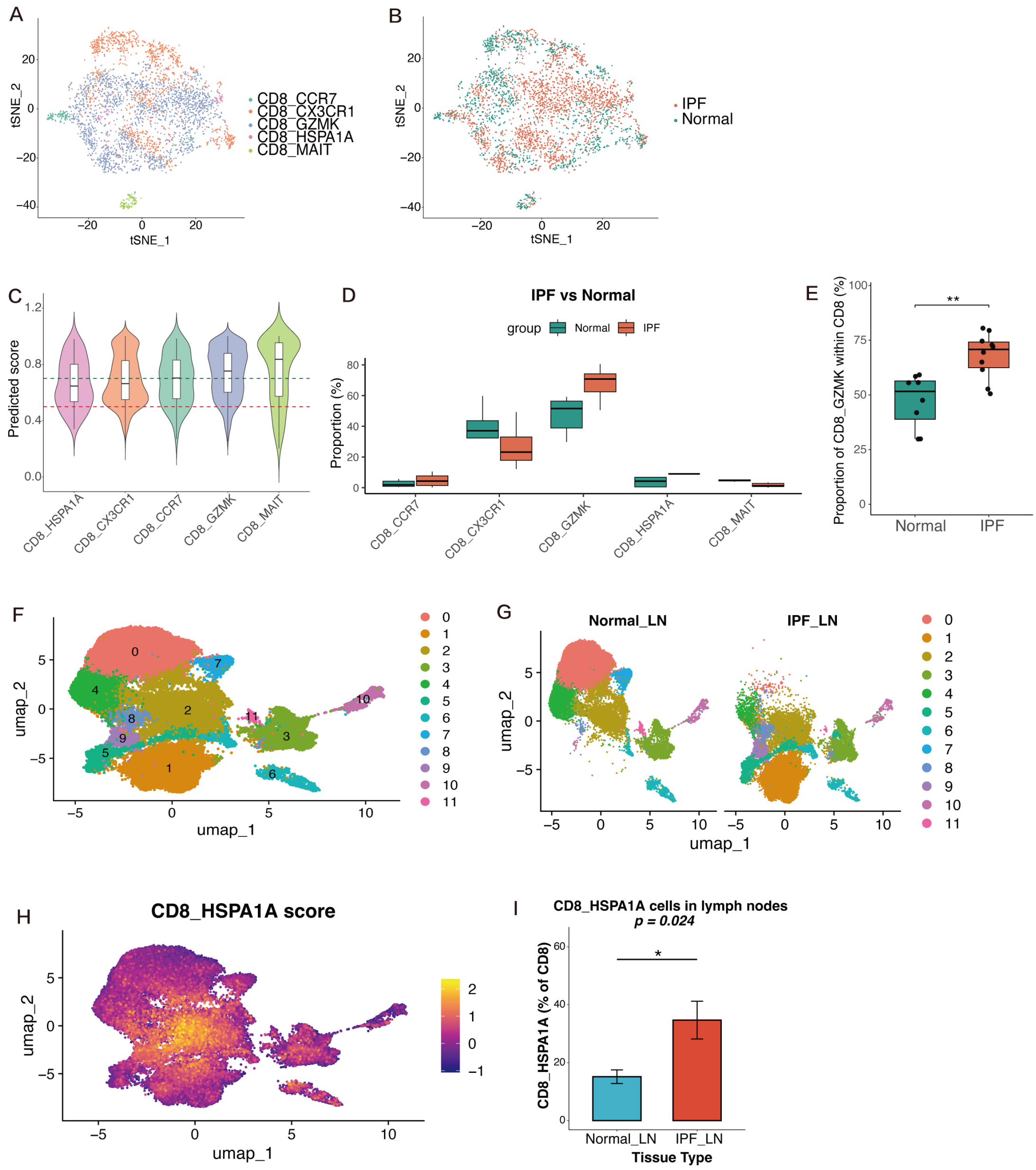

Supplementary Figure 3 Independent cohort validation of CD8_GZMK enrichment in IPF and increased CD8_HSPA1A cells in IPF lymph nodes.

A. t-SNE plot of CD8$^+$ T cells from the public scRNA-seq dataset GSE214085, coloured by predicted CD8$^+$ T cell subsets (CD8_CCR7, CD8_CX3CR1, CD8_GZMK, CD8_HSPA1A, and CD8_MAIT). **B.** t-SNE embedding coloured by different groups (IPF versus Normal). **C.** Violin plots of cell-type prediction scores for each CD8$^+$ T-cell subset, indicating high annotation confidence. **D.** Box plots showing the proportions of each CD8$^+$ T cell subset in IPF and normal control samples. **E.** Quantification of CD8_GZMK cell proportions, showing increased abundance in IPF (n=10) compared with normal controls (n=8). **F.** UMAP of integrated lymph node scRNA-seq data (normal lymph nodes from GSE131907 and IPF lymph nodes from our study), coloured by clusters. **G.** UMAP plots showing the distribution of clusters across different tissue

sources (Normal_LN versus IPF_LN). **H.** UMAP visualization showing the CD8_HSPA1A gene-signature score across the integrated lymph node dataset. **I.** Quantification of CD8_HSPA1A T cell proportions in lymph nodes, showing an increase in IPF lymph nodes (Normal_LN, n=10; IPF_LN, n=4). Statistical significance was determined using t-test or Wilcoxon test. *P< 0.05, **P< 0.01.

Supplementary Figure 4

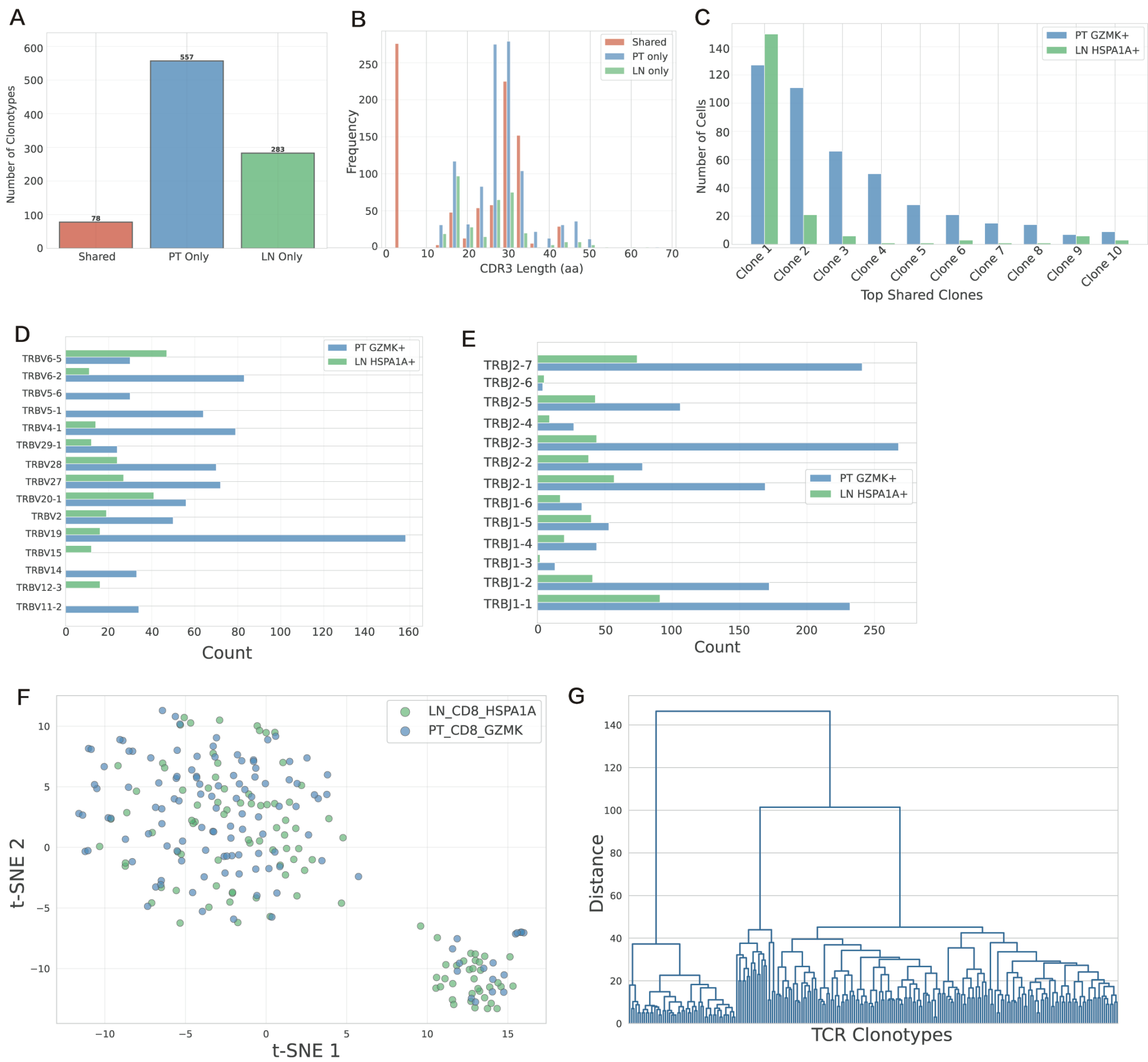

Supplementary Figure 4 Integrated TCR repertoire analysis links lymph node CD8_HSPA1A cells to lung CD8_GZMK cells.

A. Numbers of unique TCR clonotypes that are shared between lung CD8_GZMK cells and lymph node CD8_HSPA1A cells, or restricted to either tissue (PT only, LN only). **B.** Distribution of CDR3 length among shared clonotypes and tissue-restricted clonotypes. **C.** Cell counts for the top shared clonotypes across the two populations. **D–E.** TRBV (**D**) and TRBJ (**E**) gene usage in lung CD8_GZMK and lymph node CD8_HSPA1A cells. **F.** t-SNE plot of TCR clonotypes coloured by source population (lung CD8_GZMK versus lymph node CD8_HSPA1A), showing intermixing of clonotypes. **G.** Hierarchical clustering of TCR clonotypes based on sequence similarity, demonstrating overlapping TCR clusters across the two populations.

Supplementary Figure 5

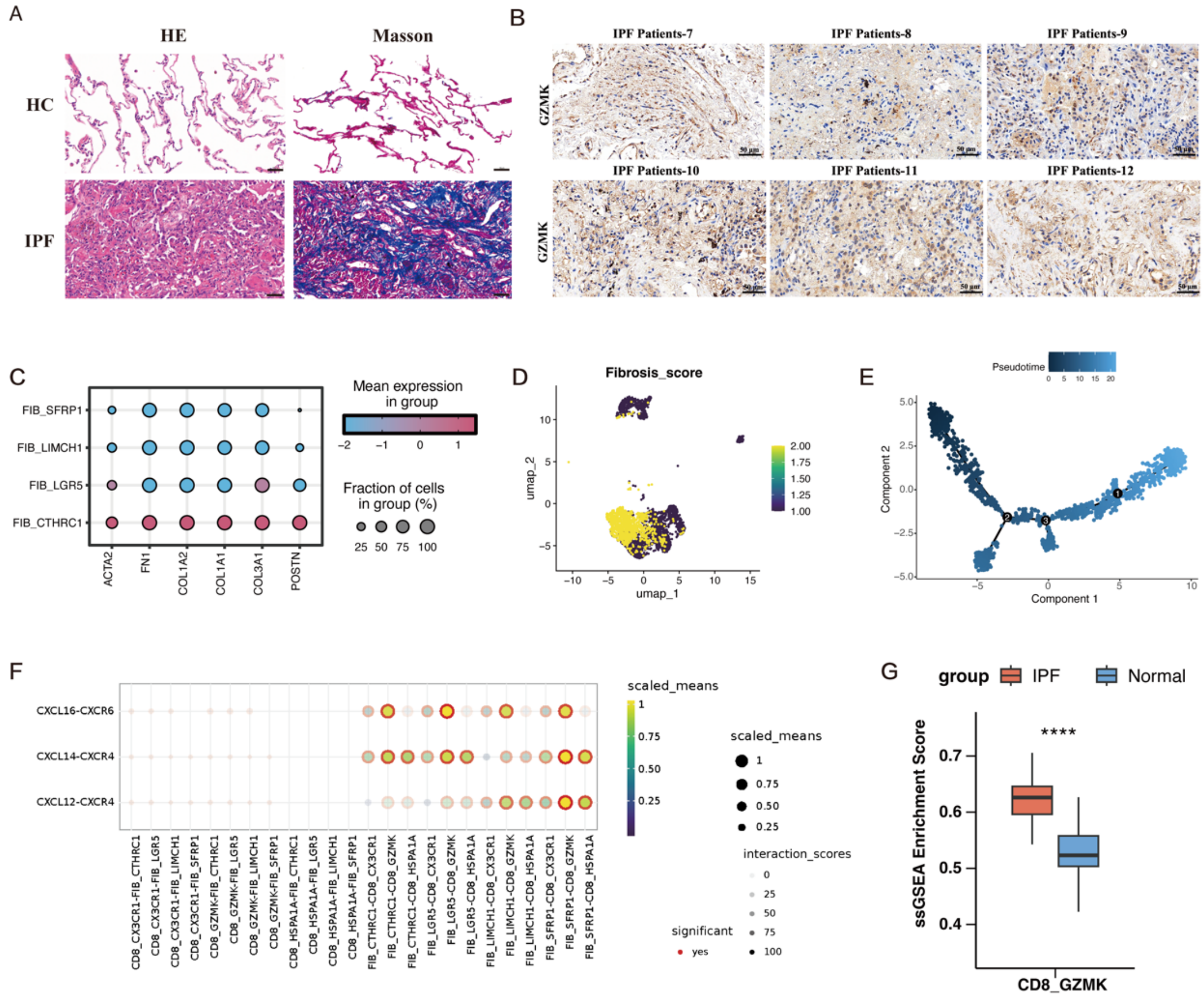

Supplementary Figure 5 CD8_GZMK cells engage profibrotic fibroblast subsets through TGF-β-mediated intercellular communication.

A. Representative haematoxylin and eosin (H&E) and Masson's trichrome staining images showing disrupted alveolar architecture and excessive collagen deposition in lung tissue of patients with IPF compared with normal lung tissue. Scale bar, 50 µm. **B.** Immunohistochemical staining of GZMK in lung tissue sections from six additional independent IPF patients. Brown signals indicate positive staining for GZMK, with variable expression levels observed across individual patients. Nuclei were counterstained with haematoxylin (blue). Scale bar, 50 µm. **C.** Dot plot displaying the expression of myofibroblast marker genes across identified fibroblast clusters. **D.** UMAP visualization showing the distribution of fibrosis score across different fibroblast clusters. **E.** The pseudotime scale is indicated by the color gradient, from dark blue (early stage) to light blue (late stage). **F.** Ligand-receptor interaction analysis of CXCL signaling between fibroblast subsets and $CD8^{+}$ T cell populations in IPF lung tissue. **G.** ssGSEA analysis comparing the infiltration scores of CD8_GZMK cells between IPF and normal tissues in the GSE213001 dataset revealed a significantly higher abundance of CD8_GZMK cells in IPF tissues.

Supplementary Figure 6

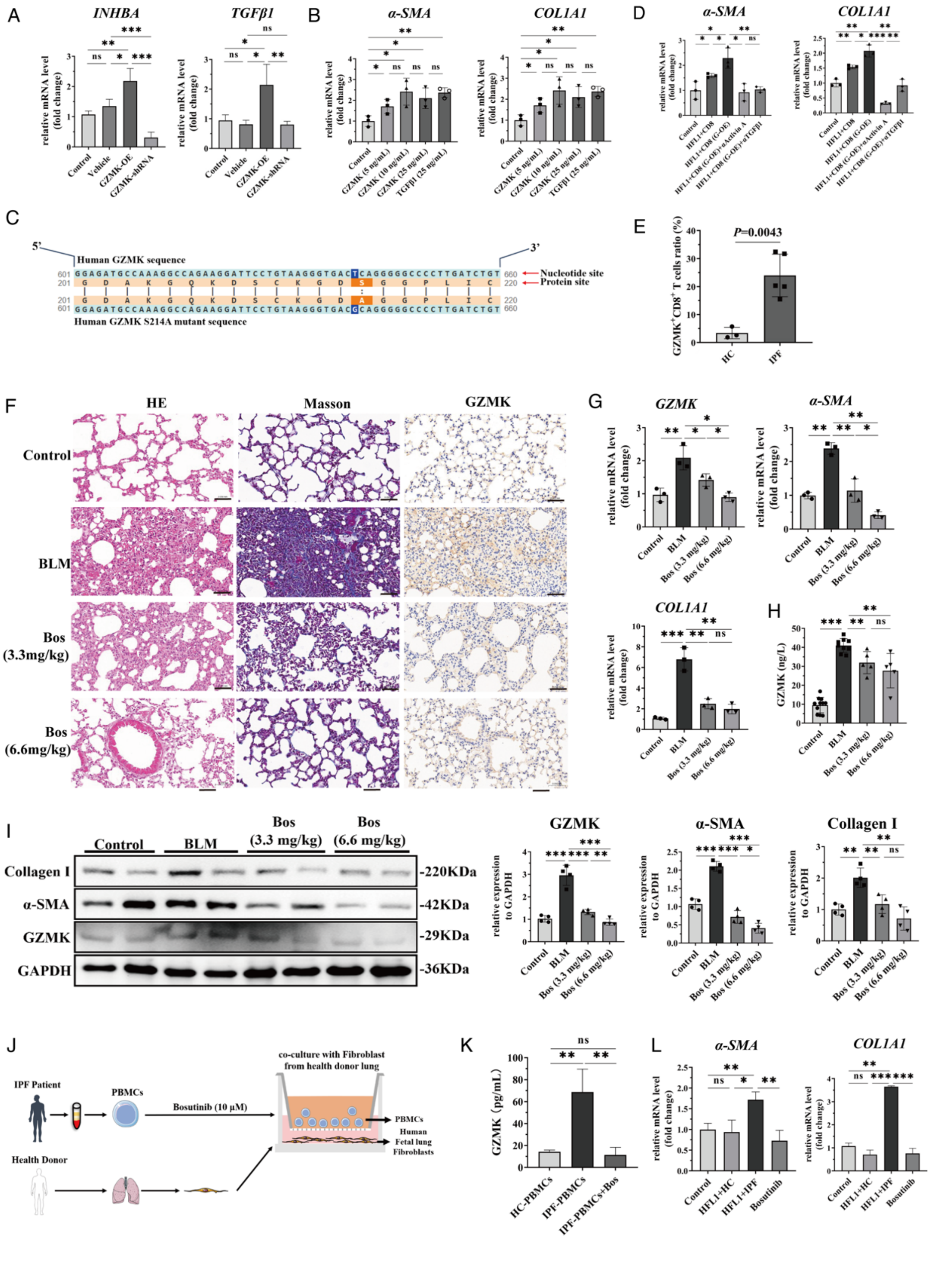

Supplementary Figure 6 GZMK inhibition by bosutinib reduces fibroblast activation and lung fibrosis.

A. qPCR analysis of *INHBA* and *TGFβ1* mRNA levels in $CD8^+$ T cells under GZMK overexpression (GZMK-OE) or GZMK knockdown (GZMK-shRNA) conditions (n=3). **B.** qPCR analysis showing the mRNA expression levels of *α-SMA* and *COL1A1* in

fibroblasts treated directly with recombinant GZMK protein at various concentrations (5, 10, or 25 ng/mL) or TGFβ1 (25 ng/mL) for comparison (n=3). **C.** The schematic diagram of S214A mutation site of GZMK. **D.** qPCR analysis of α-SMA and COL1A1 expression in HFL1 fibroblasts cocultured with GZMK-overexpressing (G-OE) $CD8^+$ T cells, treated with or without neutralizing antibodies against Activin A (αActivin A) or TGF-β1 (αTGFβ1). **E.** Flow cytometry analysis comparing the ratio of $GZMK^+$ $CD8^+$ T cells in the peripheral blood of healthy controls (HC) and IPF patients (n≥3). **F.** Bleomycin (BLM)-induced lung fibrosis mice were subsequently treated with bosutinib every 3 days (3.3 or 6.6 mg/kg, intraperitoneally) to evaluate its in vivo antifibrotic effects. Representative images of HE, Masson's trichrome, and immunohistochemical staining for GZMK in lung tissues from different groups. Scale bar, 50 μm. **G.** qPCR analysis further demonstrated that bosutinib significantly decreased the expression of *α-SMA*, *COL1A1*, and *GZMK* (n=3). **H.** Serum GZMK protein levels in the indicated treatment groups were quantified by ELISA (n≥5). **I.** Western blot analysis confirmed that bosutinib effectively decreased the protein levels of collagen I, α-SMA, and GZMK in lung tissue (n=4). **J.** Schematic diagram of GZMK inhibition in IPF patient-derived PBMC and HFL1 cell coculture chambers. **K.** ELISA analysis of GZMK levels in PBMC culture supernatants. GZMK secretion was increased in IPF-derived PBMCs and was significantly reduced by Bosutinib (Bos) treatment (n=3). **L.** qPCR analysis showing the expression levels of the fibrotic markers *COL1A1* and *α-SMA* in fibroblasts cocultured with IPF-derived PBMCs with or without treatment with bosutinib (n=3). Data are presented as mean ± standard error of the mean (SEM). Statistical significance was determined using a one-way ANOVA with multiple comparisons. *P<0.05, **P<0.01, ***P<0.001, ****P<0.0001, ns, not significant.

Supplementary table 1. Clinical Characteristics of IPF Patients for scRNA seq and TCR profiling

Characteristics	Patient 1	Patient 2	Patient 3	Patient 4
**Age**	69	65	59	66
**Sex**	Male	Male	Male	Male
**BMI**	21.1	21.7	22.3	24.8
**Smoking history**	50 years	30 years	10 years	20 years
**Oxygen therapy**	Nasal catheter	Nasal catheter	Nasal catheter	Nasal catheter
**Oxygen flow (L/min)**	3	3	3	3
**Anti-fibrotic treatment**	Pirfenidone, Nintedanib	Nintedanib	None	Pirfenidone, Nintedanib
**Lung function**				
**FEV1 (L)**	1.56	2.14	2.27	1.57
**FVC (L)**	1.82	2.16	3.12	1.69
**DLCO (mmol/min/kPa)**	2.96	NA	1.27	1.8
**Comorbidities**				
**Chronic heart disease**	None	None	Yes	None
**Diabetes**	None	None	None	None
**Hypertension**	None	None	None	Yes
**6-min walk distance (m)**	184	222	131	28
**Pulmonary artery pressure (mmHg)**	43	46	62	25

Note: NA, not available
